# Heparin-binding EGF-like growth factor via miR-126 controls tumor formation/growth and the proteolytic niche in murine models of colorectal and colitis-associated cancers

**DOI:** 10.1038/s41419-024-07126-2

**Published:** 2024-10-17

**Authors:** Yousef Salama, Shinya Munakata, Taro Osada, Satoshi Takahashi, Koichi Hattori, Beate Heissig

**Affiliations:** 1 Division of Stem Cell Dynamics, Center for Stem Cell Biology and Regenerative Medicine, The Institute of Medical Science, The University of Tokyo, 4-6-1 Shirokanedai, Minato-ku, Tokyo 108-8639 Japan; 2 An-Najah Center for Cancer and Stem Cell Research, Faculty of Medicine and Health Sciences, An-Najah National University, PO Box 7 Nablus, Palestine; 3 Department of Coloproctological Surgery, Juntendo University Faculty of Medicine, 2-1-1 Hongo, Bunkyo-Ku, Tokyo 113-8421 Japan; 4 Department of Gastroenterology, Juntendo University, Urayasu Hospital, Urayasu, Japan; 5 Division of Clinical Precision Research Platform, the Institute of Medical Science, The University of Tokyo, 4-6-1 Shirokanedai, Minato-ku, Tokyo 108-8639 Japan; 6 Center for Genome and Regenerative Medicine, Juntendo University, Graduate School of Medicine, 2-1-1 Hongo, Bunkyo-Ku, Tokyo 113-8421 Japan; 7 Department of Hematology/Oncology, The Institute of Medical Science, The University of Tokyo, 4-6-1 Shirokanedai, Minato-ku, Tokyo 108-8639 Japan; 8 Department of Bioresource Bank, Graduate School of Medicine, Juntendo University School of Medicine, 2-1-1 Hongo, Bunkyo-Ku, Tokyo 113-8421 Japan

**Keywords:** Colon cancer, Proteases

## Abstract

MicroRNAs, including the tumor-suppressor miR-126 and the oncogene miR-221, regulate tumor formation and growth in colitis-associated cancer (CAC) and colorectal cancer (CRC). This study explores the impact of the epithelial cytokine heparin-binding epidermal growth factor (HB-EGF) and its receptor epidermal growth factor receptor (EGFR) on the pathogenesis of CAC and CRC, particularly in the regulation of microRNA-driven tumor growth and protease expression. In murine models of CRC and CAC, lack of miR-126 and elevated miR-221 expression in colonic tissues enhanced tumor formation and growth. MiR-126 downregulation in colon cells established a pro-tumorigenic proteolytic niche by targeting HB-EGF-active metalloproteinase-7, -9 (MMP7/MMP9), disintegrin, and metalloproteinase domain-containing protein 9, and modulating chemokine-mediated recruitment of HB-EGF-loaded inflammatory cells. Mechanistically, downregulation of HB-EGF and EGFR in the colon suppressed miR-221 and enhanced miR-126 expression via activating enhancer-binding protein 2 alpha. Reintroducing miR-126 reduced tumor development and HB-EGF expression. Combining miR-126 reintroduction, which targets specific HB-EGF-active proteases but not ADAM17, with MMP inhibitors like Batimastat or Marimastat effectively suppressed tumor growth. This combination normalized protease expression and balanced miR-126 and miR-221 levels in developing and growing tumors. These findings demonstrate that suppressing HB-EGF and EGFR1 shifts the balance from oncogenic miR-221 to tumor-suppressive miR-126 action. Consequently, normalizing miR-126 expression could open new avenues for treating patients with CAC and CRC, and this normalization is intertwined with the anticancer efficacy of MMP inhibitors.

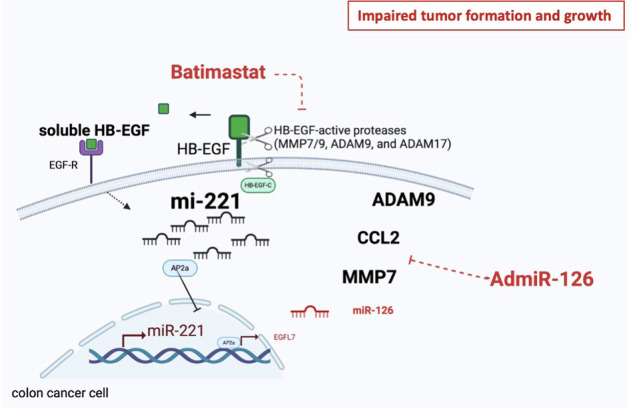

## Introduction

Colitis-associated colorectal cancer (CAC) occurs in patients with inflammatory bowel disease (IBD) [1]. CRC and CAC are controlled by microRNAs (miRNAs), small non-coding RNAs that negatively regulate their target mRNAs.

miRNAs control critical genes important for CRC [2]. High miR-221 levels in feces and colon tissues of CRC patients correlate with shorter patient survival [3, 4]. miR-221 upregulation in colon tissues of CRC patients correlated with shorter patient survival [4, 5].

miR-126 is found in intron 7 of the epidermal growth factor-like domain7 (Egfl7) gene and contributes to cancer growth [6, 7]. Reduced miR-126 levels predict poor survival and metastasis in CRC patients [8]. In contrast, high expression of miR-126 is found in IBD patients [9]. miR-126 targets the chemokine C-C-motif ligand 2 (CCL2), Egfl7, and proteases like ADAM9 or MMP7 [3, 10, 11].

The growth factor heparin-binding-epidermal growth factor-like growth factor (HB-EGF) is one of the members of the epidermal growth factor (EGF) family. The membrane-bound HB-EGF is cleaved from the cell surface by sheddases, including matrix metalloproteinase-3 (MMP-3), MMP7 [12], disintegrin metalloproteinase 9 (ADAM9), ADAM10, and its main sheddase, ADAM17/TNFα-converting enzyme [13]. ADAM17 is overexpressed in tumors and inflammation sites [13]. HB-EGF binds to the EGF receptor (EGFR)/ErbB1, promoting colorectal cancer (CRC) cell survival and proliferation (reviewed in ref. [14]) and the progression of colonic adenocarcinoma [15]. miR-221 is a downstream target of the EGFR1-RAS-RAF-MEK pathway, and miR-221 is a negative regulator of EGFR expression [16]. However, the link between the HB-EGF-EGFR pathway and miRNAs is unclear.

In this study, we hypothesized that the HB-EGF-EGFR axis and proteases that control the shedding of HB-EGF regulate the balance between the tumor-suppressor miR-126 and the proinflammatory miR-221 in cancer cells during CRC and a murine model of CAC—the so-called Azoxymethane (AOM) Dextran Sodium Sulfate (DSS) model. We observed miR-221 upregulation and miR-126 downregulation in murine models of CAC and tumor cell lines. Epithelial HB-EGF downregulation suppressed miR-126 expression. Restoration of miR-126 expression and cotreatment with MMP inhibitors (MMPi) normalized expression of HB-EGF and HB-EGF-active proteases in the murine CAC model.

We discovered that MMPi treatment and gene silencing of HB-EGF-active proteases enhanced miR-126 expression and that MMPi-driven anti-proliferative effects required cellular miR-126 expression. MMPis, such as Batimastat (Batim) contributed to the normalization of HB-EGF expression in the colon epithelium. We propose combining AdmiR-126 OE and MMPi for CRC suppression.

## Material and methods

### Mice

8–12-week-old male C57BL6 mice were purchased from Japan SLC Inc. (Hamamatsu, Japan). The institutional Animal Care and Use Committee of Juntendo University School of Medicine, Tokyo, Japan, and AnNajah University approved the animal procedure protocols. All animal experiments complied with the National Institutes of Health guide for the care and use of Laboratory animals (NIH Publications No. 8023, revised 1978).

### AOM DSS model

C57BL/6 mice were injected intraperitoneally (i.p.) with 12.5 mg/kg AOM (Wako) dissolved in physiological saline. Seven days later, 2% DSS (ICN Biomedical molecular weight = 36,000–50,000 Da) was given in the drinking water over five days, followed by 16 days of regular water for three cycles. We sacrificed animals on day 84 or 80. Examiners counted visible tumor counts in a blinded fashion.

In the AdmiR-126 rescue experiment, AOM DSS or non-AOM DSS mice were injected i.v. with adenoviral injection using AdMock or AdmiR-126 OE (1 × 10^9^ plaque-forming units/mouse/injection) preparations on days 20 and 35 after the initial AOM injection. On day 0, mice were randomly divided into the different treatment groups (*n* = 7/treatment group). Two adenoviral injections were chosen because the innate inflammatory response following Ad vector administrations limits the duration of transgene expression in C57/BL6 mice. We, therefore, decided on the Ad injections on days 20 and 35.

### S.C. colon cancer murine model

We injected Mock and miR-126 OE CMT93 cells (2.5 × 10^7^/0.2 ml of PBS)subcutaneously (s.c.) in the inguinal region of mice and the same number of cells seven days later in the same injection location. To meet statistical requirements, 28 mice were randomly divided into four groups (7 per group) to minimize experimental error. After 7 days, mice were randomly divided into the different treatment groups. The MMPi batimastat (Sigma, USA) was used at indicated concentrations in vitro and 30 mg/kg intraperitoneally (i.p., starting injections from day ten after tumor inoculation). The number of mice was 7 per treatment group. The MMPi marimastat was injected i.p. daily (15 mg/kg body weight) starting from day 10. Marimastat-injected mice were sacrificed on day 25. Then, we weighed the extracted tumors and/or generated tumor cell suspensions to isolate CD11b/F4/80 cells in a two-step labeling and isolation procedure: first, with anti-CD11b-coated beads, and then using F4/80 Macs beads (Milteney Biotec).

### Cell lines and primary cells

Mouse rectal cancer cells (ATCC: CCL-223) were cultured in DMEM (high glucose) with L-glutamine and phenol red (Wako, Japan) containing 10% fetal bovine serum (FBS) and 1% penicillin/streptomycin (P/S; Nacalai Tesque Inc, Japan). Human HT-29 (ATCC; HTB-38) cells were cultured in McCoy’s 5a Medium Modified, Wako, Japan) containing 10% FBS and 1% P/S.

### Cell culture

HT-29 cells (1 × 10^5^ cells/well) or CMT93 cells (1 × 10^5^ cells/well) were seeded in six-well plates (TPP, Switzerland), kept overnight, and transfected by using Lipofectamine RNAiMAX (Invitrogen). In addition, we cultured transfected cells or cells cotreated with rec. HB-EGF (Cat no. 100-47, Peprotech) for an additional 24 hr in the presence or absence of chemicals/inhibitors. Cultures were performed with *n* = 6–7 per treatment group, as indicated in the figure legends. We counted viable cells after Trypan blue exclusion to determine cell proliferation. In addition, we collected cells for cDNA synthesis.

### Quantitative reverse transcriptase-polymerase chain reaction

Total RNA was isolated from colon tissues treated by AOM DSS using a Nucleospin RNA plus kit (Takara Bio, Otsu, Japan). RNA extraction and cDNA generation were described elsewhere [17]. We used miRNeasy Mini Kit (Qiagen, Germany) for miRNA extraction. We performed reverse transcription using the Mir-XTM miRNA First-Strand Synthesis Kit (cat no 638313, Takara, Japan). Data are presented as a relative fold change to controls according to the comparative Ct method (2−ΔΔCt). Gene expression was normalized for all qPCR results to b-actin mRNA expression unless otherwise mentioned. Analysis was performed using 3 different cell samples in triplicate. The levels of expression of miR-126 were normalized to U6 using the 2−ΔΔCT method.

**Table:** 

Forward	Reverse
mMMP9 5'-AGACGACATAGACGGCATCC-3'	5'-TCGGCTGTGGTTCAGTTGT-3'
mADAM28-F- 5'-GCTATAGTGATCCAGCGCCA-3'	5'-TGGTGGATGGTAGCCTCTGA-3'
mHB-EGF 5'-TCTTCTTGTCATCGTGGGACT-3'	5'-CACGCCCAACTTCACTTTCT-3'
mAP2a 5'-AGTTCACAGTTTTTCAGCTATGGA-3'	5'-GCGCTGGTGTAGGGAGATT-3'
mMMP7 5'-TAATTGGCTTCGCAAGGAGA-3'	5'-AAGGCATGACCTAGAGTGTTCC-3'
mADAM9 5'-TTTCTCCGGCAGTGAGTACA-3'	5'-GCATTGAAGCTTTCCACACA-3'
mTIMP-1 5'-ATTCAAGGCTGTGGGAAATG-3'	5'-CTCAGAGTACGCCAGGGAAC-3'
mTIMP-2 5'-GCATCACCCAGAAGAAGAGC-3'	5'-GGGTCCTCGATGTCAAGAAA-3'
mTIMP-3 5'-CCGAGGCTTCAGTAAGATGC-3'	5'-CCTCTCCACAAAGTTGCACA-3'
mCCL2 5'-CATCCACGTGTTGGCTCA-3'	5'-ATCATCTGCTGGTGAATGAGT-3'
mADAM17 5'-GTACGTCGATGCAGAGCAAA-3'	5'-AAACAGAACAGACCCAACG-3'
miR-126 5'-CATTATTACTTTTGGTACGCGCTGT-3'	
miR-221 5'-AGCTACATTGTCTGCTGGGTTTC-3'	
mb-actin 5'-CTAAGGCCAACCGTGAAAAG-3'	5'-ACCAGAGGCATACAGGGACA-3'
hHB-EGF 5'-TGGGGCTTCTCATGTTTAGG-3'	5'-CATGCCCAACTTCACTTTCTC-3'
hAP2a 5'-AACATGCTCCTGGCTACAAAA-3'	5'-AGGGGAGATCGGTCCTGA-3'
hMMP7 5'-GACATCATGATTGGCTTTGC-3'	5'-TCTCCTCCGAGACCTGTCC-3'
hADAM9 5'-TCCCCCAAATTGTGAGACTAA-3'	5'-TCCGTCCCTCAATGCAGTAT-3'
hADAM17 5'-TCATTGACCAGCTGAGCATC-3'	5'-CGCAGGAAAGGGTTTGATAA-3'
hb-actin 5'-CCAACCGCGAGAAGATGA-3'	5'-CCAGAGGCGTACAGGGATAG-3'

### Cell culture and siRNA transfection

We plated cells at a concentration of 2 × 10^5^ cells/well in a six-well plate. We transiently transfected cells with Lipofectamine RNAiMAX (Invitrogen) one day later. We used the following siRNA targeting sequences:

Si-miR-221 5′-GAA CCU GGC AUA CAA UGU ATT-3′

miR-126 KO plasmid (kindly provided by Ai Kotani, Tokai University.

Si-Ctrl: 5′-GCUCCACAGAGUAUACCUU-3′

Si-HB-EGF#1: 5′-GGA GAG GAG GUU AUG ACU UTT-3′

Si-HB-EGF#2 5′-CGU GGUGAUGCUGAAGCUCUUUCU-3′

Si-EGFR#1: 5′-GGAUGUGAAGUGUGGCCAU-3′

Si-EGFR#2: 5′-UCGGGAGCAUUUGGCACAGUGUAUA-3′

Si-MMP7: 5′-CGGUACUGUGAUGUACCCUACCUAU-3′

Si-ADAM9: 5′-CCCGACUCCUUUAUCCUAUGACUUA-3′

Si-ADAM17: 5′-ACAUUUCAGGCACUCGGGACAGAGU-3′

si-ADAM28, 5′-UUCAGCCACAGUCUUCCAC-3′

### Plasmid construction

#### miR-126 cloning

We extracted genomic DNA from T17b cells using the genomic DNeasy Extraction Kit (Qiagen, Germany). The genomic sequence of the segments in primary (pri)-miR-126 was amplified by genomic PCR using PrimeStar polymerase (Takara, Japan). The primers used for PCR were 5′-GGGGCTCGAGCTGGCTCCTTGCCTGGTGGA-3’ (forward) and 5′-TTTTCCCGGGTGGCCACTGCCACAGCTGTGGGG -3’ (reverse). PCR products were purified using a PCR purification kit (Qiagen) and sequenced. We cloned the purified genomic PCR product into the XhoI and SmaI sites of a mammalian Lentiviral expression vector, LV-EF-L3T4-IRES2-EGFP, and the constructed vectors were sequenced to confirm the insertion of miR-126.

#### AP2a cloning

We amplified the mouse AP2a coding sequence from brain tissue cDNA. We used the primers 5′-GGGCTCGAGTCCATGAAAATGCTTT-GGAAACTGACGGA-3 and 5-CCCGATATCTCACTTTCTGTGTTTCTCTTCTTT-GTCA-3’ to amplify mouse AP2a by genomic PCR with PrimeSTAR polymerase (Takara, Japan). Then, we inserted the purified fragment into the XhoI and EcoR V sites of the eukaryotic expression vector LV-EF-L3T4-IRES2-EGFP.

#### miR-221 cloning

We extracted genomic DNA from CMT93 cells using the genomic DNeasy Extraction Kit (Qiagen, Germany). Genomic PCR amplified the genomic sequence using PrimeSTAR polymerase (Takara, Japan). The primers used for PCR were 5′-CTCGAGGACTGTTGGTTTTCTTTTCCTTGT-3′ and 5′-CCCGGGTCAATGATAAACTCCACTGGTT-3′. We inserted the purified fragment into the XhoI and SmaI sites of the eukaryotic expression vector LV-EF- L3T4-IRES2-EGFP. All cloned plasmids were validated following transformation and amplification in E. coli DH5α competent cells by double restriction enzyme digestion, separated by 1% PAGE, and sequenced by Sanger sequencing (FASMAC, Japan).

### Plasmid transfection

Cells (2 × 10^5^ per well in a 6-well plate) were transfected with plasmids using Lipofectamine 3000 (Invitrogen). Twenty-four hours after transfection, we replaced the transfection mixture with complete media and used the cells in the experiments.

### Adenovirus preparation

We recently reported expanding adenoviral vectors expressing human miR-126 and AdNull containing no transgene (Mock) [17]. We used the following kit: Cat. #6170, Takara, Japan. Null Adenovirus (AdNull) has the SRa promoter and SV40 poly(A) signal.

### Lentivirus generation

As recently described for EGFL7, we designed miR-126 shRNAs using the webpage http://sirna.wi. mit.edu/home.php [6, 17].

### Tissue staining

Histopathologic and immunohistochemical analyses of mouse colon tissues. Resected mouse colon tissues were fixed in 4% formalin neutral buffer solution for paraffin embedding or snap-frozen in Tissue-Tek Ornithine Carbamyl Transferase compound. Paraffin-embedded sections were stained with H&E. Tissue microarray slides containing adenocarcinoma (Adeno-Ca) and non-tumor adjacent colon tissue samples (Cat no. T8235790D-2, BioChain, USA), including 32 matched malignant/non-malignant pairs were purchased from BioChain (USA). Tissues were stained using the human HB-EGF antibody (clone: G-11, Santa Cruz). After staining with an appropriate secondary HRP-labeled antibody, 3,3’-diaminobenzidine-tetrahydrochloride (IVIEW Roche Tissue Diagnostics, Indianapolis, IN, USA) addition resulted in the visualization of the peroxidase activity.

### Western blotting analysis

Cell lysates (2–50 μg proteins) were generated as described previously [6, 17] Membranes (Millipore, Immobilon) were probed with one of the following primary antibodies (1 µg/ml) overnight at 4 °C: C-terminal cytoplasmic domain of HB-EGF of mouse origin (Santa Cruz Biotech, sc-1414), ADAM9 precursor (Santa Cruz Biotech, sc-377233), MMP7 (Santa Cruz Biotech, sc-515703), huADAM117 (Santa Cruz Biotech, sc-6416), p-ERK (Cell Signaling, #4370), CCL2 (Santa Cruz Biotech, sc-52701); AP2a (Cell Signaling, #3215); mouse b-actin (Cell Signaling, #4967). Membrane staining with appropriate secondary antibodies conjugated with horseradish peroxidase (Nichirei, rabbit-HRP, or goat-HRP) was followed by membrane development using the ECL Plus detection system (Amersham Life Science, RPN2132). We analyzed the images using the image analyzer Image-Quant LAS4000 (GE Healthcare, Uppsala, Sweden).

### Webserver timer

TIMER is a comprehensive webserver and resource for analyzing the correlation between gene expression and immune infiltrates across diverse cancer types (https://cistrome.shinyapps.io/timer/) [18].

### Statistical analysis

Data are the mean ± standard error of the mean (SEM). *p* values < 0.05 indicate statistically significant differences. We compared multiple groups using the one-way analysis of variance (ANOVA) and analyzed two groups using the Student *t* test.

## Results

### HB-EGF is upregulated in colonic tissues of AOM DSS-treated mice

**Fig. 1:**
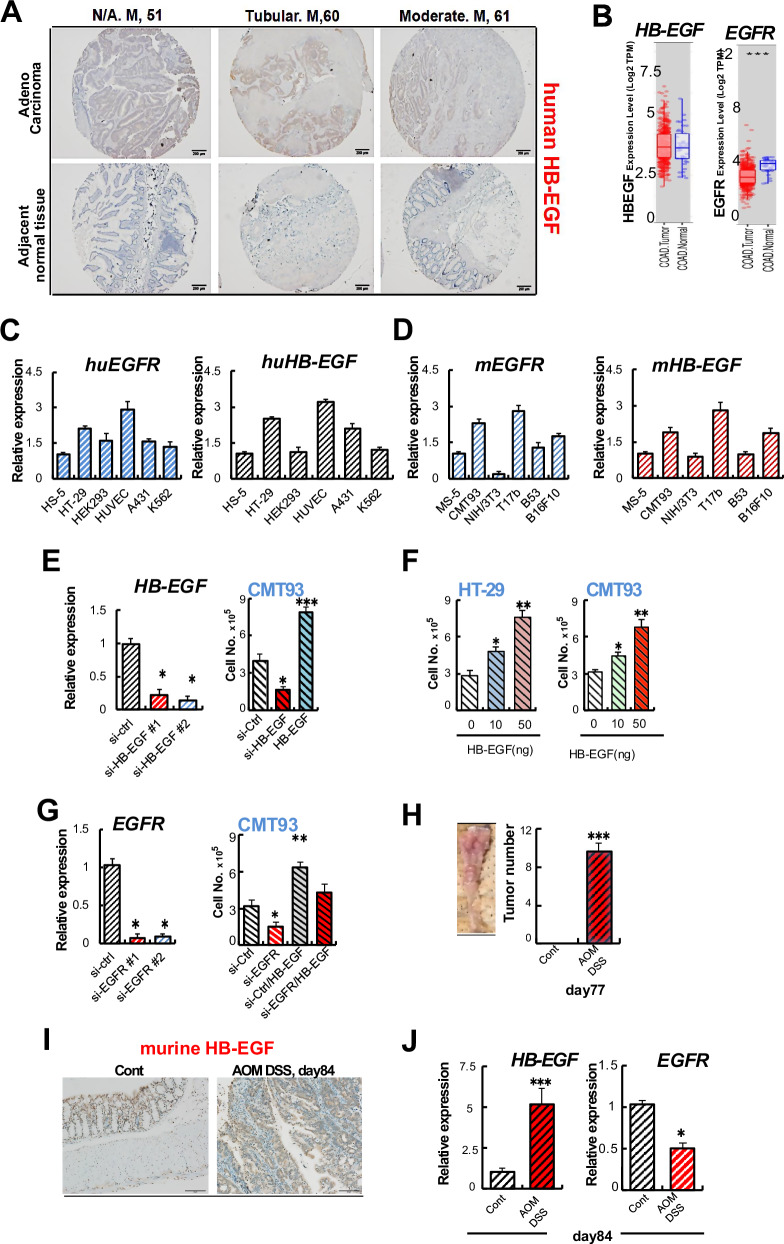
High colonic HB-EGF during colitis-associated cancer (CAC). **A** Representative, selected immunohistological images stained for human HB-EGF of tumor and adjacent non-tumor tissues extracted from a tissue array for human colon cancer: scale bar, 200 μm. Figure S1A shows all array images. **B** Timer determined human *HB-EGF* and *EGFR* expression in human colon adenocarcinoma (COAD) from the TCGA database. **C**, **D** Fold change in human (**C**) and mouse (**D**) *EGFR* and *HB-EGF* expression in indicated cells, including the human (HT-29) and murine colon cancer cell lines (CMT93), compared to that in HS-5 (for human) and MS-5 (for mouse) cells. Other cell lines included the Human embryonic kidney cells (HEK293), epidermoid carcinoma in the skin (A431), K562 cells and the mouse NIH3T3 cells, embryonal endothelial progenitor cells (T17b), plasma cell line (B53), and melanoma cells (B16F10). **E** Left panel: fold change of *HB-EGF* expression in si-HB-EGF compared to CMT93 cells transfected with scrambled siRNA (si-ctrl) as determined by qPCR. Right panel: cell proliferation of si-ctrl CMT93 and si-HB-EGF cells treated with/without rec. HB-EGF. Cells were counted 24 hours after plating (*n* = 6/group). **F** Human HT-29 and murine CMT93 cells were cultured with/without rec. HB-EGF. Cells were counted 24 hours later (*n* = 6/group). **G** Left panel: fold change in *EGFR* expression in si-EGFR compared to si-ctrl cells as confirmed by qPCR. Right panel: Proliferation of si-EGFR and si-ctrl CMT93 cells 24 h after the addition of rec. HB-EGF using cell counts (*n* = 6/group). **H** The CAC model was established by injecting AOM on day 0 i.p, followed by three cycles of 2% DSS in drinking water for mice (*n* = 8/group; upper panel). Left panel: Representative macroscopic image showing tumor formation in the distal colon of AOM/DSS mice after 13 weeks. Right panel: Number of tumors >2 mm at day 77. **I** Representative images of HB-EGF-stained colonic tissues of AOM DSS and non-AOM DSS-treated mice retrieved at day 84: scale bars, 50 μm. **J** Fold change in *HB-EGF* and *EGFR* expression in colonic tissue extracts of AOM DSS mice compared to the expression in control tissues retrieved on day 84 as determined by qPCR (*n* = 3/group). The expression of all genes is normalized to the endogenous reference b-actin and presented as a relative fold change to controls. Mean ± SEM, unpaired Student’s *t* test. *p < 0.05, **p < 0.01; *** *p* < 0.001.

Here, we aimed to assess the role of microRNA-126 (miR-126) in colon cancer tumor growth, especially by studying its correlation with HB-EGF. To that end, we analyzed HB-EGF expression in human colon adenocarcinoma. *HB-EGF* expression varied between tumor and normal adjacent from human colon adenocarcinoma (COAD) patients as determined by immunohistochemical analysis and RNA-seq data (Fig. 1A, B). As reported previously [19], lower *EGFR* expression was found in tumors compared with adjacent normal colon tissues of COAD patients (Fig. 1B). The analysis of various cell lines revealed the highest *EGFR* and *HB-EGF* expression in endothelial cells (human umbilical vessel cells/HUVEC) or T17b cells), followed by the humans HT-29 and mouse CMT93 colorectal cancer cells compared to stromal cell lines human HS-5 and mouse MS-5 cells as determined by quantitative PCR (qPCR) (Fig. 1C, D).

We corroborated reports by others on the proliferation-stimulating effects of HB-EGF. The addition of recombinant (rec.) HB-EGF enhanced CMT93 and HT-29 cell proliferation, while HB-EGF knockdown (KD) by siRNA (si-HB-EGF) slowed down tumor growth (Fig. 1E, F). Furthermore, we confirmed that the blockade of EGFR using si-EGFR and treatment with neutralizing antibodies against EGFR prevented HB-EGF-mediated tumor growth (Fig. 1G).

Next, we determined EGFR and HB-EGF expression in a murine colon carcinogenesis model, the AOM DSS model. The combination of the carcinogen AOM and the exposure to the inflammatory agent DSS given to mice recapitulates the aberrant crypt foci to adenoma to carcinoma sequence occurring in human CAC. Tumors had developed by day 84 in AOM DSS-treated mice (Fig. 1H). We analyzed the tumor tissues of mice by day 84. Tumors growing in AOM DSS-treated mice compared to control colon tissues showed high *HB-EGF* by immunohistochemistry (Fig. 1I) or low *EGFR* but high *HB-EGF* expression by qPCR (Fig. 1J). Our data indicated high HB-EGF expression in colon tissues of AOM DSS-treated mice. In contrast, EGFR expression was low in the AOM DSS model.

### HB-EGF and EGFR knockdown enhances miR-126 expression in colon cancer cells

**Fig. 2:**
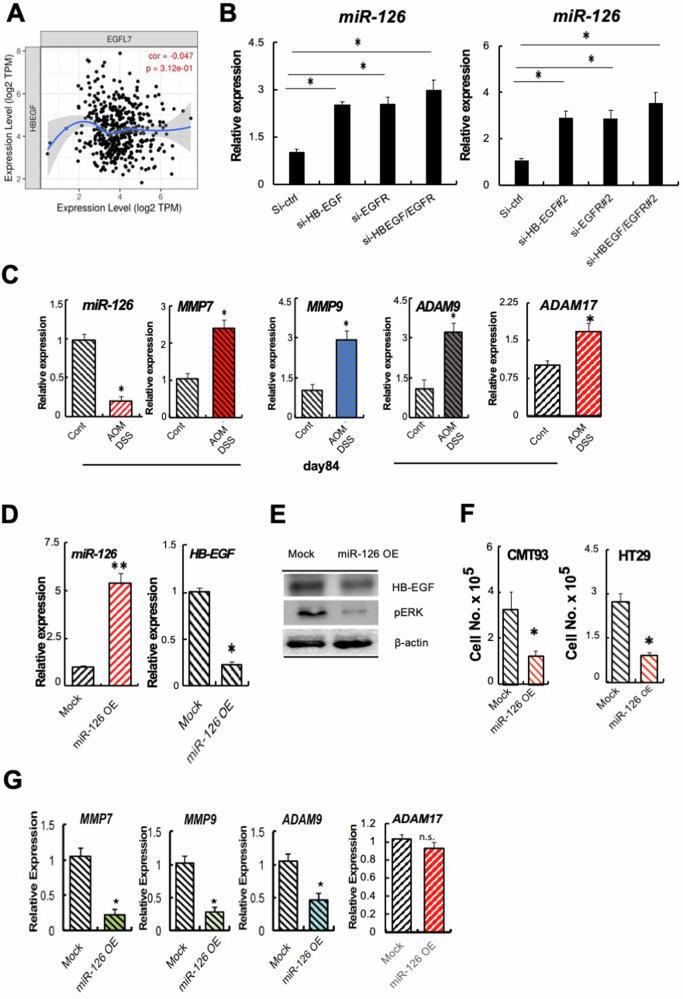
HB-EGF-EGFR silencing restores miR-126 expression in CRC cells. **A** Spearman’s rank-correlation for *EGFL7* and *HB-EGF* mRNA expressions analyzed in colon tissues of patients with COAD is shown using scatter plots (*n* = 457 patients; Spearman’s rho value and estimated statistical significance). **B** Fold change in *miR-126* expression in si-HB-EGF, si-EGFR, or si-HB-EGF/si-EGFR CRC cells compared to expression in si-ctrl cells by qPCR. The left panel shows results using CMT93 cells, and the right panel presents data using HT-29 cells. **C** Fold changes in *miR-126*, *MMP7*, *MMP9*, *ADAM9*, and *ADAM17* expression in colonic tissue extracts of AOM DSS mice compared to the expression in control non-AOM DSS tissues retrieved on day 84 as determined by qPCR (*n* = 3/group). **D** Fold change in *miR-126* and *HB-EGF* expression of miR-126 OE compared to Mock CMT93 cells determined after 24 h (*n* = 6/group). **E** Representative immunoblots for HB-EGF, phosphorylated ERK1/2, and b-actin using miR-126 OE and Mock CMT93 cell lysates. B-actin control and HB-EGF or phosphorylated ERK1/2 were run on separate gels. Samples for Fig. 2E and samples from a study by Salama et al. [34] were run on the same gel/Western blot to detect b-actin. **F** The proliferation of miR-126 OE and Mock CMT93 and HT-29 cells was determined after 24 h. (*n* = 6/group). **G** Fold change in *MMP7*, *MMP9*, *ADAM9*, and *ADAM17* expression in miR-126 OE compared to Mock cells as determined by qPCR (*n* = 3/group). Normalization of genes to U6 for miR-126 or b-actin for other genes. Mean ± SEM, unpaired Student’s *t* test. *p < 0.05, **p < 0.01.

*HB-EGF* inversely correlates with *Egfl7*, the host gene of miR-126 expression in melanoma [20]. Like in melanoma, we found an inverse correlation between the expression of *HB-EGF* and *Egfl7* in tumor tissue of COAD patients (Fig. 2A; Spearman’s rho value −0.0473445, *p* = 0.3118836; *n* = 457; data analysis using Timer [18]).

To test the effects of HB-EGF and/or its primary receptor EGFR on miR-126 expression, we generated HB-EGF or EGFR single and double knockdown (KD) cells using two siRNAs for each target gene in two different CRC cell lines (Fig. 2B). The knockdown of HB-EGF and EGFR using two different siRNAs for each gene target was confirmed using qPCR (data not shown). *miR-126* expression increased by ~2–3-fold in si-HB-EGF, si-EGFR cells, and si-HB-EGF/si-EGFR double KD CMT93 cells (Fig. 2B), suggesting that HB-EGF and/or EGFR signaling modulate miR-126 suppression.

Because HB-EGF was high in AOM DSS colonic tissues, we hypothesized that an inverse expression pattern between HB-EGF and miR-126 might exist in tumor tissues of AOM DSS mice. Indeed, we found low *miR-126* expression in colonic tissues on day 84 in colonic tissues of AOM DSS-treated compared to control mice (Fig. 2C). miR-126 inhibits metalloproteinase 9 (ADAM9) expression [21], a protease that promotes HB-EGF cleavage. We found high expression of HB-EGF-associated proteases (Fig. 2C), including *ADAM9*, *ADAM17, MMP7*, and *MMP9* in colon tissues of AOM DSS but not non-AOM DSS mice.

To determine whether miR-126 directly could alter CRC growth and HB-EGF-associated protease expression, we overexpressed miR-126 (OE) in miR-126 low-expressing CMT93 cells (Fig. 2D). miR-126 OE reduced HB-EGF expression, impaired ERK phosphorylation (Fig. 2D, E), and diminished tumor cell growth in human and murine miR-126 OE, but not control cells (Fig. 2F).

In support of our in vivo data, miR-126 OE CRC cells expressed less *MMP7*, *MMP9*, *and ADAM9* but not *ADAM17* than Mock cells (Fig. 2G). We confirmed that miR-126 OE downregulated CCL2 expression in colon cancer cells in vitro (Supplementary Fig. 2A). In contrast, the expression of the three endogenous MMPi called tissue inhibitors of metalloproteinase *(TIMP)-1*, *-2*, and *-3* [22] was similar in miR-126 OE compared to Mock cells (Supplementary Fig. 2B), indicating that miR-126 restoration in CRC cells suppressed proteases like MMP7 and -9 but not their endogenous inhibitors.

### HB-EGF and EGFR enhance miR-221 but suppress miR-126 expression

**Fig. 3:**
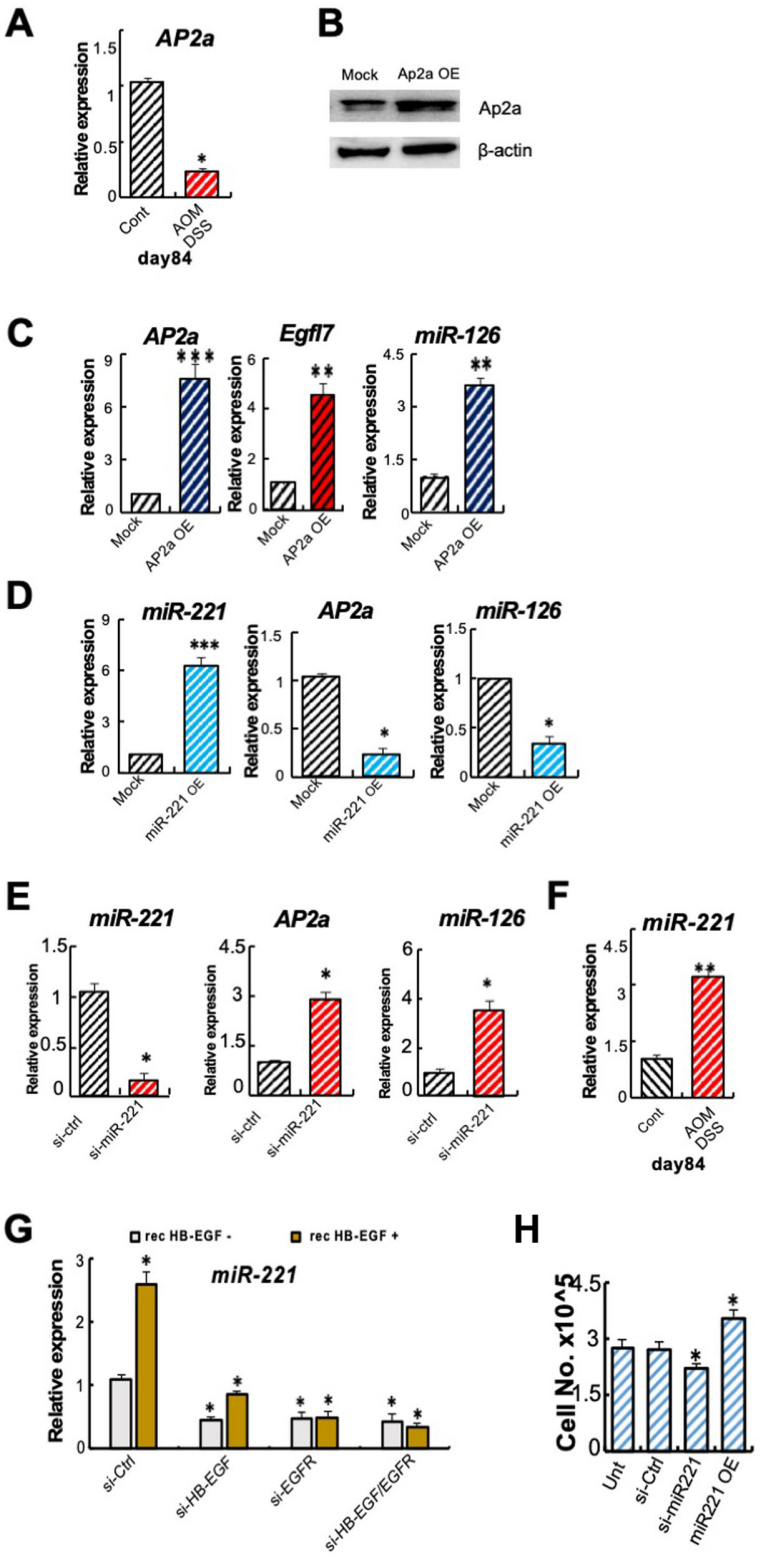
HB-EGF and EGFR control miR-221 expression that targets AP2a expression. **A** Fold change in *AP2a* expression in colon tissues from AOM DSS mice compared to untreated control mice at day 84 (*n* = 3/group) by qPCR. **B** Representative immunoblot for AP2a and the loading control b-actin in AP2a OE and Mock cells. **C** Fold change in *AP2a*, *Egfl7*, and *miR-126* expression in AP2a OE compared to Mock CMT93 cells as determined by qPCR (*n* = 3/group). **D** and **E** Fold change in *miR-221*, *AP2a*, *and miR-126* expression in miR-221 OE (**D**) and miR-221 (**E**) silenced CMT93 compared to Mock cells (*n* = 3/group). **F** Fold change in *miR-221* expression in colon tissues of AOM/DSS mice compared to untreated control mice at day 84 (*n* = 3/group) by qPCR. **G** Fold change in *miR-221* expression in si-HB-EGF, si-EGFR, or si-HB-EGF/si-EGFR CMT93 cells treated with or without rec. HB-EGF compared to the gene expression in si-ctrl CMT93 by qPCR. **H** Cell proliferation of untreated si-Ctrl, si-miR-221, and miR-221 OE cells as determined by cell counting (*n* = 6/group). Mean ± SEM, unpaired Student’s *t* test. *p < 0.05 and **p < 0.01.

Our data indicated that HB-EGF and EGFR controlled miR-126 expression in CRC cells. To further elucidate the mechanism underlying this regulation, we searched for factors that influence Egfl7 expression. The transcription factor AP2a is a tumor repressor in CRC [23] and binds to the Egfl7 promoter. We detected low *AP2a* expression in colonic tissues of AOM DSS compared to control mice (Fig. 3A). AP2a OE enhanced *Egfl7* and *miR-126* expression in CRC but not Mock cells (Fig. 3B, C).

A reverse expression pattern of miR-126 and miR-221 has been reported, with AP2a linking both miRs. AP2a was directly targeted by miR-221&222 while, on the other hand, it enhances miR-126&126* expression [20]. As expected, miR-221 OE impaired, while miR-221 knockdown enhanced *AP2a* and *mi-R126* expression in CRC cells (Fig. 3D, E). Furthermore, *miR-221* expression was increased in colon tissues of AOM DSS but not in control mice (Fig. 3F).

Because miR-221 expression is linked to EGFR activation in glioblastoma [16], we next examined miR-221 expression in HB-EGF and EGFR single KD or combined KD CRS cells. Individual HB-EGF or EGFR KD and the combined HB-EGF/EGFR KD reduced *miR-221* expression compared to si-Ctrl CRC cells (Fig. 3F). The addition of recombinant/soluble HB-EGF enhanced *miR-221* expression in control and si-HB-EGF cells but failed to do so in si-EGFR or co-silenced si-HB-EGF/si-EGFR cells (Fig. 3G). Functionally, miR-221 OE enhanced, while si-miR-221 reduced cell proliferation (Fig. 3H). These results indicated soluble HB-EGF and EGFR signaling enhances miR-221 expression in CRC cells.

### HB-EGF-active protease knockdown and MMPi treatment impair CRC proliferation with *miR-221* down- and *miR-126* upregulation

**Fig. 4:**
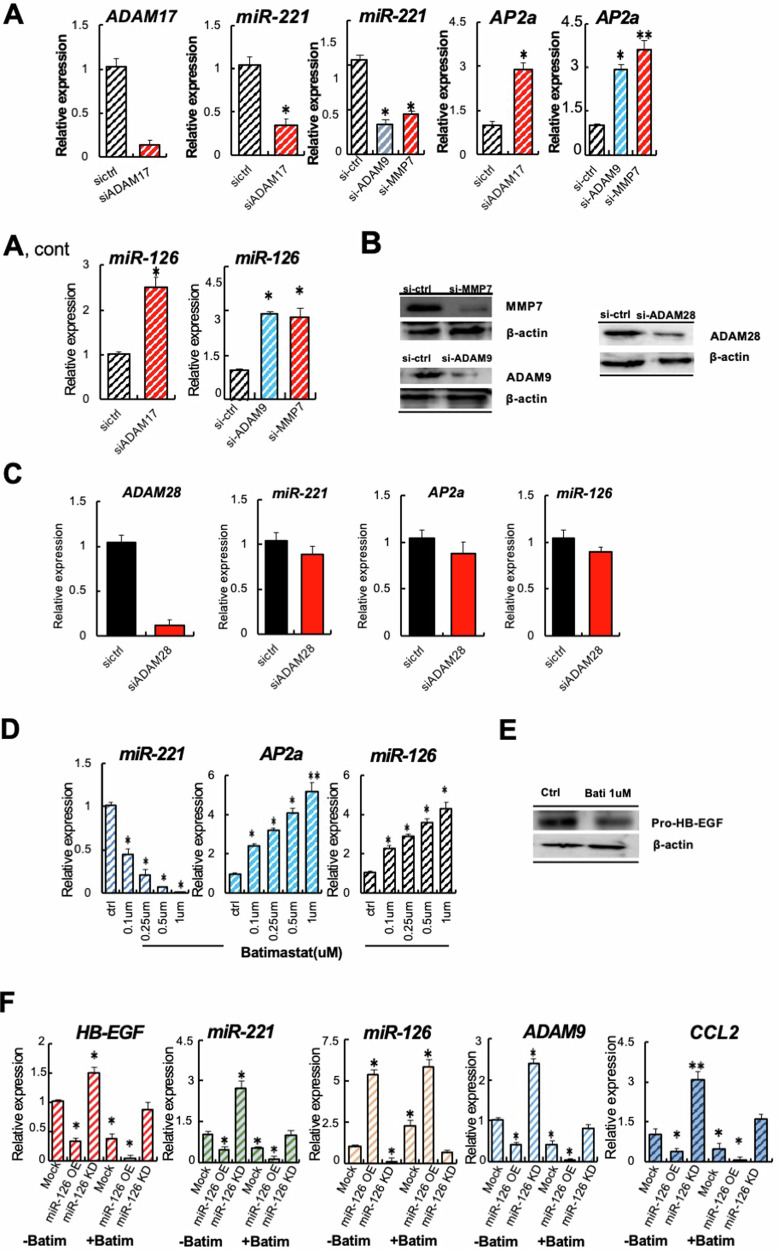
Silencing of MMP7, ADAM9, and ADAM17 or MMPi treatment enhances miR-126 expression, while miR-126 restoration impairs their expression. **A** Fold change in *ADAM17*, *miR-221*, *AP2a*, or *miR-126* expression in si-ADAM17, si-ADAM9, and si-MMP7 cells compared to expression in si-ctrl CMT93 cells (*n* = 3/group). **B** Representative immunoblots for MMP7, ADAM9, ADAM28 and b-actin in si-ctrl, si-MMP7, or si-ADAM9 and si-ADAM28 CMT93 cells. **C** Fold change in *ADAM28*, *miR-221*, *AP2a*, or *miR-126* expression in si-ADAM28 CMT 93 cells (*n* = 3/group). **D** Fold change in *miR-221*, *AP2a*, and *miR-126* expression in wild-type CMT93 cells treated with indicated concentrations of batimastat compared to expression in untreated ctr cells (*n* = 3/group). **E** Representative immunoblots for HB-EGF and b-actin in cell lysates from CRC cells treated without or with batimastat. **F** Fold change in *HB-EGF*, *miR-221*, *miR-126*, *ADAM9*, and *CCL2* expression in Mock, miR-126 OE, and miR-126 KD cells treated with/without batimastat (*n* = 3/group). B-actin was run on a separate gel from the other proteins for all immunoblots. Mean ± SEM unpaired Student's *t* test. *p < 0.05, **p < 0.01, ***p < 0.001.

Proteolytic cleavage of HB-EGF promotes the generation of HB-EGF-C that, after interaction with PLZF delocalization, causes miR-221&222 upregulation. Next, we tested if HB-EGF-active proteases highly expressed in AOM DSS mice’s colonic tissues alter miR-221, AP2a, or miR-126 expression. Gene silenced si-ADAM17, si-ADAM9, and si-MMP7 CRC, but not control cells showed low *miR-221* and high *miR-126* and *AP2a* expression (Fig. 4A, B). Knockdown of ADAM28, a protease related to fertilization and neurogenesis but not linked to HB-EGF processing, did not alter any of the mentioned genes (Fig. 4C). These data indicated that ADAM17, ADAM9, and MMP7 downregulated miR-126 and enhanced miR-221 expression in CRC cells.

The broad-band MMPi Batimastat also targets the expression of the HB-EGF-active proteases *MMP7 and ADAM9* but is less active in reducing *ADAM17* expression in CMT93 cells (Supplementary Fig. 2C). Batimastat reduced *miR-221*, increased *AP2a* and *miR-126* expression dose-dependent (Fig. 4D), and reduced pro-HB-EGF expression in CRC cells (Fig. 4E). We had established that HB-EGF KD impaired miR-221 and upregulated miR-126 expression (see Figs. 3E and 2B). The single protease knockdown and MMPi inhibitor treatment regulate the balance between miR126 versus miR-221 and AP2a expression in CRC cells.

Our data shows that miR-126 OE and MMPi treatment enhance miR-126 and decrease miR-221 expression in CRC cells, inhibiting tumor growth. However, their HB-EGF-active protease target profiles differ. Batimastat, but not miR-126 OE, suppresses the key HB-EGF-active proteases. Thus, we propose that combined miR-126 OE and MMPi treatment could more effectively suppress tumor growth by normalizing the expression of colonic epithelial proteases and HB-EGF expression.

Indeed, the gene expression analysis of cultured CRC cells unveiled that Batimastat effectively suppressed *HB-EGF*, *miR-221*, *ADAM9*, and the chemokine ligand 2 (*CCL2*),—another miR-126 target gene and known macrophage chemokinetic factor expression in miR-126 OE and Mock, but less in miR-126 KD cells (Fig. 4F). Batimastat suppressed ADAM9 on the protein level (Supplementary Fig. 2D). The most effective suppression of suppression of HB-EGF, miR-221, ADAM9, and CCL2 occurred in CRC miR-126 OE cells cotreated with Batimastat (Fig. 4F).

### MMPis block tumor growth in miR-126 expressing, but not miR-126 KD cells

**Fig. 5:**
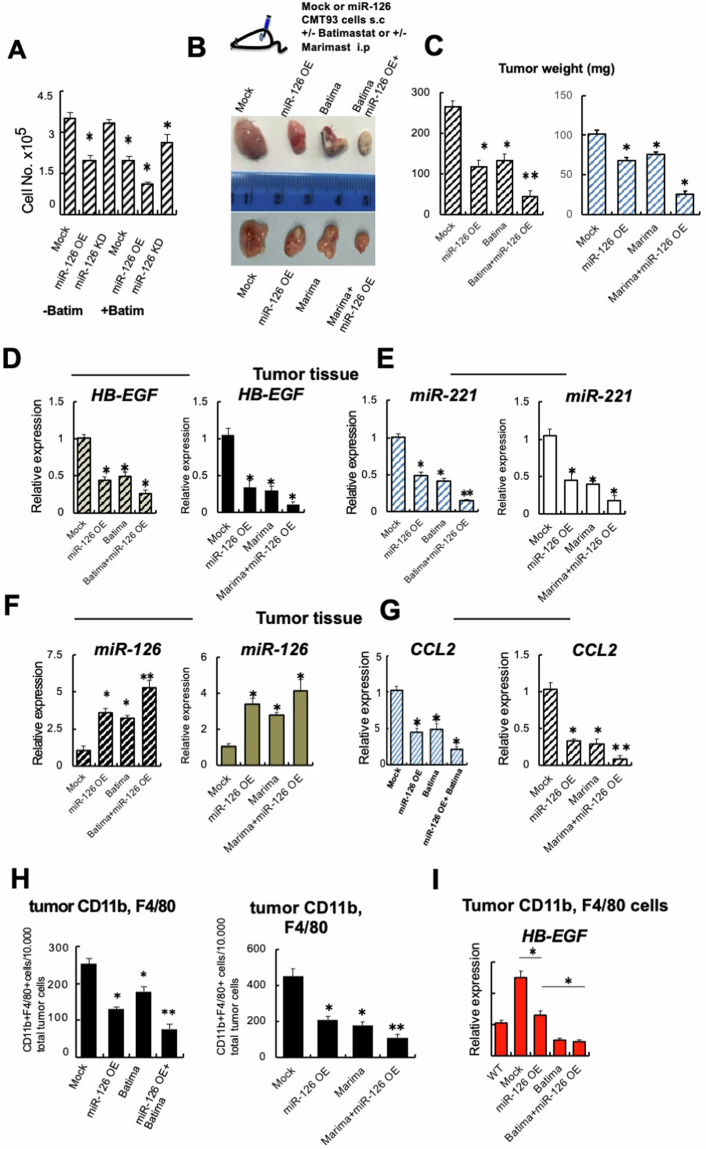
miR-126 restoration in CRC cells enhances the anti-tumor effect of MMPi and impairs intratumoral myeloid cell influx in a syngenic tumor model. **A** Cell proliferation of miR-126 OE, KD, or Mock cells treated with/without Batimastat 24 hours after cell plating (*n* = 3/group). **B** Upper panel: Experimental design of orthotopic CRC model: Injection of murine miR-126 OE and Mock CMT93 cells s.c. into C57/BL6 mice, followed by treatment with or without Batimastat or Marimastat starting from day 10 (*n* = 7/group). Lower panel: macroscopic tumor images were taken on day 25 post-inoculation. **C** Tumor weight on day 25 (*n* = 7/group). **D**–**G** qPCR analysis of *HB-EGF* (**D**), *miR-221* (**E**), *miR-126* (**F**), and *CCL2* (**G**) expression in miR-126 OE and Mock tumor tissues of indicated MMPi treatment groups (left panels Batimastat and right panels Marimastat) compared to nontreated groups (*n* = 3/group). Results are presented relative to expression in Mock cell-derived tumors. **H** CD11b + F4/80+ cell numbers in crushed tumor tissues of mice injected with Mock, miR-126 OE CMT93 cells and treated with/without indicated MMPi (Batimastat or Marimastat) on day 25 (*n* = 3/group) after two-step MACS isolation. The absolute number of double-positive cells per 10,000 total tumor cells is shown. **I** Fold change in *HB-EGF* expression in CD11b^+^F4/80^+^ cells isolated from tumors of Mock or miR-126 OE mice treated with or without batimastat compared to peripheral blood CD11b^+^F4/80^+^ cells of non-tumor mice. Normalization of genes to an endogenous reference (U6 for miR-126 or miR-221, or b-actin for other genes). Mean ± SEM, unpaired Student’s *t* test. *p < 0.05, **p < 0.01, ***p < 0.001.

Next, we tested CRC proliferation in miR-126 OE and KD cells treated with/without Batimastat. Batimastat most effectively blocked proliferation in miR-126 OE and to some extent in Mock cells but was ineffective in miR-126 KD cells, suggesting that the anti-tumor effect of Batimastat was highest in miR-126 expressing, but not miR-126 KD cells (Fig. 5A).

Encouraged by the efficient growth suppression in vitro, we test the in vivo anti-tumor effects of a combined treatment using miR-126 OE strategies and the cotreatment with two MMPi inhibitors (Batimastat and Marimastat) in an orthotopic syngeneic murine CRC model using CMT93 cells. miR-126 OE and Mock cells were injected subcutaneously (s.c.) into C57/Bl6 mice. The MMPis were injected intraperitoneally daily in groups of mice (Fig. 5B). Mock CRC cells grew in all mice. Batimastat-treated Mock tumor-carrying mice showed a ~50% reduction in tumor size, while tumor size reduction after Marimastat treatment was ~30% (Fig. 5B, C). miR-126 OE tumors were smaller than Mock tumors. However, the tumors were smallest in animals cotreated with MMPis (Batimastat or Marimastat), suggesting that the anti-tumor effect of MMPis was highest in miR-126 expressing cells.

The tumor cell analysis of mice injected with miR-126 OE CRC cells and treated with MMPi revealed the lowest *HB-EGF*, *miR-221, CCL2*, and the highest *miR-126* expression (Fig. 5D–G).

Combined miR-126 OE with MMPi treatment impaired myeloid cell recruitment and reduced cancer and myeloid cell *HB-EGF* expression.

Because the chemokine CCL2 regulates myeloid cell recruitment and low CCL2 expression was found in miR-126 OE tumor tissues, we examine the number of CD11b and F4/80 expressing myeloid cells in tumors. Tumors grown in Mock CRC-injected mice had the highest number of myeloid cells (CD11b+ F4/80+) (Fig. 5F), while ~50% fewer myeloid cells/10,000 tumor cells infiltrated in tumors of Mock cell-injected mice treated with MMPi or in tumors of miR-126 OE-injected mice (Fig. 5H). We detected the lowest number of myeloid cells in miR-126 OE tumors of mice treated with MMPi (Fig. 5H). These data suggest combining miR-126 OE with MMPi treatment strategies impaired CD11b/F4/80+ cell recruitment into tumors and blocked tumor growth.

Given that macrophages are a rich source of HB-EGF, we also analyzed HB-EGF expression in tumor-associated macrophages. Of interest, the analysis of tumor-derived macrophages isolated from miR-126 OE tumors in mice treated with Batimastat showed the lowest HB-EGF (Fig. 5I), suggesting that the combined treatment of miR-126 reintroduction and Batimastat suppresses HB-EGF expression in tumor and inflammatory cells in the tumor niche that might indirectly in a paracrine fashion contribute to tumor growth suppression.

### Batimastat plus miR-126 reintroduction prevents tumor formation during CAC

**Fig. 6:**
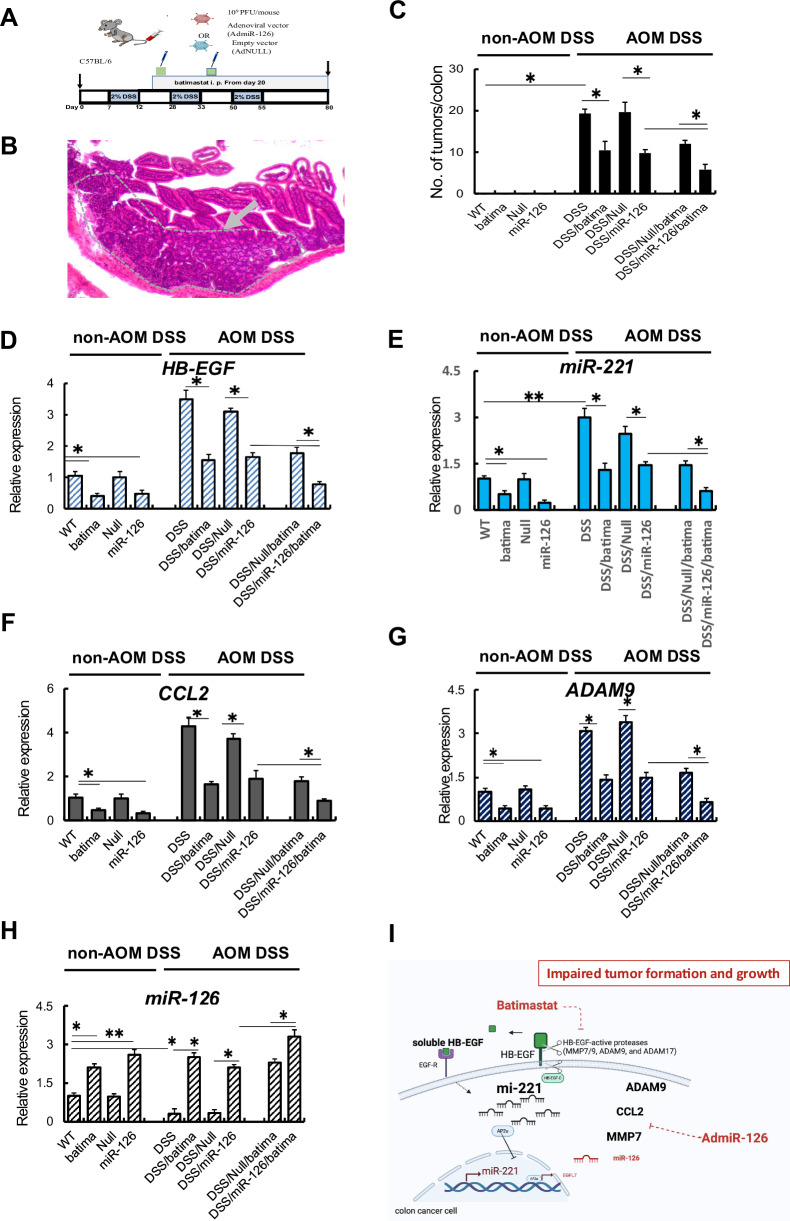
MMPi and miR-126 OE suppress tumor formation in the AOM DSSS model. **A** Experimental setting: mice receiving AOM and DSS (AOM DSS mice) and mice not receiving AOM and DSS (non-AOM DSS mice) received AdmiR-126 or AdNull adenovirus injections and were cotreated daily with/without Batimastat from day 20. **B** A Hematoxylin & Eosin-stained colon section in AOM/DSS-treated mice shows a typical neoplastic lesion indicated by arrow and dashed lines. Scale bar: 100 μm. **C** Macroscopically detectable tumor numbers per colon of indicated groups of mice (*n* = 7/group). **D**–**H** Colon tissues retrieved on day 80 were examined for *HB-EGF* (**D**), *miR-221* (**E**), *CCL2* (**F**), *ADAM9* (**G**), and *miR-126* (**H**) expression by qPCR. Relative to expression in colon tissues of mice with AOM/DSS (*n* = 3/group) normalized to b-actin. Mean ± SEM, *p* values from unpaired Student’s *t* test or ANOVA. *p < 0.05, **p < 0.01. **I** During the malignant transformation, inflammatory and tumor cells upregulate proteases, including ADAM17, resulting in the cleavage of HB-EGF to generate soluble HB-EGF and HB-EGF-C. Soluble HB-EGF via EGFR signaling and HB-EGF-C enhances miR-221 expression. In turn, miR-221 targets AP2a, a transcription factor that controls the transcription of Egfl7 and miR-126 located in intron 7 of the Egfl7 gene. The miR-126-miR-221 imbalance in tumor cells establishes a pre-cancerous, proteolytic environment and “unleashes” the expression of HB-EGF, proteases, and chemokines like CCL2. This destabilized environment promotes leukocyte influx and protease release. The anti-tumor function of MMPis, like Batimastat or Marimastat, was most effective in miR-126-expressing cells. The reintroduction of miR-126 combined with MMP inhibition blocks HB-EGF expression, induces miR-126 expression and reduces the leukocyte influx It normalized the protease imbalance, and effectively suppressed HB-EGF expression in colonic tissues.

To this point, we showed that miR-126 OE and MMPi treatment suppressed tumor growth in CRC murine tumor models, partly by reducing HB-EGF-active proteases, impairing miR-221, and enhancing miR-126 expression. Given that miR-221, miR-126, and proteases also play a role in CAC, we asked whether the observed synergistic anti-proliferative effects of miR-126 OE and Batimastat influenced tumor (adenoma) formation in AOM DSS-treated mice. We triggered miR-126 OE by injecting adenovirus carrying miR-126 (AdmiR-126) or an empty vector (AdNull) on days 20 and 35 into AOM DSS or non-AOM DSS-treated mice. In addition, groups of mice were co-injected with/without batimastat (Fig. 6A). The body weight in the treatment groups did not significantly change throughout the experiment (Supplementary Fig. 2E). A histological image of a typical adenoma formed within the mucosa that had developed in AdNull-treated AOM DSS mice treated without Batimastat is shown in Fig. 6B.

Scoring of intestinal lesions revealed no tumor in C57/Bl6 mice (non-AOM DSS) treated with AdNull, AdmiR-126, or Batimastat (Fig. 6C). Administration of AdmiR-126 or batimastat diminished tumor development in AOM DSS compared to untreated or AdNull AOM DSS mice. Mice that had received batimastat and AdmiR-126 injection developed the lowest number of tumors (Fig. 6C), indicating that miR-126 introduction suppressed tumor formation in this colon carcinogenesis model.

Finally, we analyzed the gene expression on colonic tissues derived from non-AOM DSS and AOM DSS-treated mice 80 after the start of the experiment. Cotreatment of batimastat with AdmiR-126 showed the most robust decrease in the expression of *HB-EGF*, *miR-221, CCL2, ADAM9 (*Fig. 6D–G), *MMP7*, *MMP9* (Supplementary Fig. 2F–G), but increase in *miR-126* expression (Fig. 6H) compared to untreated controls. AdmiR-126 or batimastat as a single therapy were less effective in suppressing *HB-EGF*, *miR-221*, *CCL2*, and proteases and in inducing *miR-126* expression in colon tissues of AOM DSS-treated animals (Fig. 6D–H).

Although AOM DSS enhanced colonic *ADAM17* expression, neither Batimastat alone nor in combination with AdmiR-126 did *ADAM17* expression change (Supplementary Fig. 2H). Colonic tissues of AOM DSS showed a decrease in *EGFR* expression (Supplementary Fig. 2I). Batimastat given alone or in combination with AdNull or AdmiR-126, but not AdmiR-126 treatment given alone reinstated *EGFR* expression in colonic tissues of AOM DSS mice similar to the expression levels found in colon tissues of healthy mice.

MiR-126 reinstatement combined with MMP inhibition blocked HB-EGF expression in colonic tissues and suppressed tumor formation in a CRC tumor model and the AOM DSS colon carcinogenesis model. Furthermore, this treatment normalized the proteolytic niche, resulting in protease expression similar to healthy controls and the suppression of pro-oncogenic miR-221 expression.

## Discussion

In this study, we established a link between HB-EGF and/or EGFR signaling and the action of the tumor-suppressor miR-126 and the oncogene miR-221 in colon tumor formation and growth using murine models of CAC and CRC. We confirmed previous findings that miR-126 expression is downregulated in tumor tissues during CAC and CRC in mice [24, 25]. Reintroducing miR-126 reduced but did not block tumor development and growth and suppressed miR-221 expression. miR-221 demonstrated oncogenic potential in melanoma and, as shown here in colorectal cancer cells, targeted the tumor repressor AP2a, which regulates Egfl7 and miR-126 expression in colon epithelial cells (Fig. 6I).

The miR-126 reconstitution or MMPi treatment achieved partial tumor suppression, but combining both treatments was necessary to suppress tumor growth and normalize protease expression in CRC and CAC mice.

Proteases that enhance HB-EGF cleavage are associated with tumor progression in CRC and CAC. miR-126 overexpression reduced the expression of HB-EGF-active proteases ADAM9 and MMP7, but not ADAM17. miR-126 reconstitution partially restored the protease balance in CRC and CAC tumor and myeloid cells that control the expression status of HB-EGF and EGFR. However, miR-126 does not target all proteases deregulated during the malignant process. Cotreatment of AdmiR-126 and batimastat impaired tumor generation and growth in a colonic carcinogenesis model. We suggest that data presented here might contribute to a “Renaissance of MMPis for CAC and CRC” by considering the cellular miR-126 cancer cell status as a measure for dose adjustments. We propose that identifying the miR-126 status might help select CRC patients where MMPis like Batimastat and Marimastat are effective, potentially allowing for a dose reduction of MMPis that will reduce side effects [26].

Combining miR-126 reintroduction with MMPi treatment normalized the proteolytic niche and reinstated the miR-126-miR-221 balance as found in normal tissues (Fig. 6I). Aside from epithelial cells, immune cells (macrophages, mast cells, T cells) can also express HB-EGF, where they promote migratory functions. Additionally, cotreatment with batimastat and AdmiR-126 reduced HB-EGF expression in myeloid cells, a primary HB-EGF source. Tumors of mice injected with miR-126 OE CRCs and Mock cells and cotreated with batimastat had fewer CD11b+F4/80+ myeloid cells. Fewer CD11b+F4/80+ tumoral cells, correlating with decreased CCL2 expression, a key myeloid cell chemoattractant.

Earlier studies demonstrated that Batimastat inhibits human colon tumor growth and spread in a patient-like orthotopic model in nude mice [27]. MMP7 correlates with the grade of ulcerative colitis-associated dysplasia or carcinoma. It controls tumor growth and metastasis [28].

Our study demonstrated that Batimastat upregulates miR-126 expression in cultured cells and CAC mice’s colonic tissues and impairs the expression of HB-EGF-active proteases, including MMP7/9, ADAM9, and ADAM17. ADAM17 is the major sheddase of HB-EGF. ADAM17 KD enhanced miR-126 and decreased miR-221 expression. It is worth mentioning that the delivery of anti-miR-221 targets the endogenous ADAM17 inhibitor TIMP-3, thereby further increasing ADAM17 activity [29]. The deregulated miR-221-miR-126 circuit ultimately augments ADAM17 activity and HB-EGF cleavage.

A first-in-human study evaluated an antisense oligonucleotide targeting miR-221 for treating patients with advanced solid tumors and multiple myeloma (https://clinicaltrials.gov/ct2/show/NCT04811898). LNA-i-miR-221 reduces cell viability, induces apoptosis in vitro, and impairs tumor growth in preclinical in vivo models of CRC [30]. Our study is the first to report that the MMPis Batimastat and Marimastat downregulate miR-221 and upregulate miR-126 expression in colon epithelial cells. Batimastat’s anti-tumor effect depends on the miR-126 expression status of tumor cells. Batimastat was ineffective in controlling tumor growth in miR-126 KD colon cancer cells in vitro.

HB-EGF or EGFR KD enhanced miR-126 and impaired miR-221 expression in colon cancer cells. We found increased miR-221 expression in CRC and CAC mice tumors, corroborating reports on its role in tumorigenesis [31]. miR-132 downregulates HB-EGF in IgE-stimulated mast cells [32]. and is upregulated in AOM DSS-induced CAC, alleviating CAC severity by suppressing macrophage infiltration and proinflammatory cytokines. In light of our data, it is interesting to speculate that miR-132, by targeting HB-EGF, contributes to the ameliorating effects of 2,3,7,8-tetrachlorodibenzo-p-dioxin in the AOM DSS model [33].

Developing treatment protocols that include the systemic delivery of miR-126 with protease inhibitors might be a novel strategy for colon cancer treatment, especially in individuals where chronic inflammation participated in tumor development.

## Supplementary information

Original western blots_All

FigureS1_2

## Data Availability

Data are presented in the main manuscript.

## References

[1] Li J, Ma X, Chakravarti D, Shalapour S, DePinho RA. Genetic and biological hallmarks of colorectal cancer. Genes Dev. 2021;35:787–820. 34074695 10.1101/gad.348226.120 PMC8168558

[2] Ghafouri-Fard S, Hussen BM, Badrlou E, Abak A, Taheri M. MicroRNAs as important contributors in the pathogenesis of colorectal cancer. Biomed Pharmacother. 2021;140:111759. 34091180 10.1016/j.biopha.2021.111759

[3] Jalil AT, Abdulhadi MA, Al-Ameer LR, Abbas HA, Merza MS, Zabibah RS, et al. The emerging role of microRNA-126 as a potential therapeutic target in cancer: a comprehensive review. Pathol Res Pract. 2023;248:154631. 37393667 10.1016/j.prp.2023.154631

[4] Cai K, Shen F, Cui J-H, Yu Y, Pan H-Q. Expression of miR-221 in colon cancer correlates with prognosis. Int J Clin Exp Med. 2015;8:2794–8. 25932237 PMC4402884

[5] Yau TO, Wu CW, Dong Y, Tang CM, Ng SSM, Chan FKL, et al. microRNA-221 and microRNA-18a identification in stool as potential biomarkers for the non-invasive diagnosis of colorectal carcinoma. Br J Cancer. 2014;111:1765–71. 25233396 10.1038/bjc.2014.484 PMC4453736

[6] Salama Y, Heida AH, Yokoyama K, Takahashi S, Hattori K, Heissig B. The EGFL7-ITGB3-KLF2 axis enhances survival of multiple myeloma in preclinical models. Blood Adv. 2020;4:1021–37. 32191808 10.1182/bloodadvances.2019001002 PMC7094020

[7] Heissig B, Salama Y, Takahashi S, Okumura K, Hattori K. The multifaceted roles of EGFL7 in cancer and drug resistance. Cancers (Basel). 2021;13:1014. 10.3390/cancers13051014 PMC7957479 33804387

[8] Selven H, Busund LR, Andersen S, Bremnes RM, Kilvær TK. High expression of microRNA-126 relates to favorable prognosis for colon cancer patients. Sci Rep. 2021;11:9592. 33953222 10.1038/s41598-021-87985-3 PMC8100289

[9] Yarani R, Shojaeian A, Palasca O, Doncheva NT, Jensen LJ, Gorodkin J, et al. Differentially expressed miRNAs in ulcerative Colitis and Crohn’s disease. Front Immunol. 2022;13:865777. 10.3389/fimmu.2022.865777 PMC9208551 35734163

[10] Jia AY, Castillo-Martin M, Bonal DM, Sanchez-Carbayo M, Silva JM, Cordon-Cardo C. MicroRNA-126 inhibits invasion in bladder cancer via regulation of ADAM9. Br J Cancer. 2014;110:2945–54. 24823697 10.1038/bjc.2014.245 PMC4056059

[11] Felli N, Felicetti F, Lustri AM, Errico MC, Bottero L, Cannistraci A, et al. miR-126&126* restored expressions play a tumor suppressor role by directly regulating ADAM9 and MMP7 in melanoma. PloS One. 2013;8:e56824. 23437250 10.1371/journal.pone.0056824 PMC3578857

[12] Yu WH, Wu E, Li Y, Hou HH, Yu SC, Huang PT, et al. Matrix metalloprotease-7 mediates nucleolar assembly and intra-nucleolar cleaving p53 in gefitinib-resistant cancer stem cells. iScience. 2020;23:101600. 33089100 10.1016/j.isci.2020.101600 PMC7559243

[13] Saad MI, Jenkins BJ. The protease ADAM17 at the crossroads of disease: revisiting its significance in inflammation, cancer, and beyond. FEBS J. 2023;291:10–24. 10.1111/febs.16923 37540030

[14] Halder S, Basu S, Lall SP, Ganti AK, Batra SK, Seshacharyulu P. Targeting the EGFR signaling pathway in cancer therapy: What’s new in 2023? Expert Opin Ther Targets. 2023;27:305–24. 37243489 10.1080/14728222.2023.2218613 PMC10330690

[15] Zhou J, Ji Q, Li Q. Resistance to anti-EGFR therapies in metastatic colorectal cancer: underlying mechanisms and reversal strategies. J Exp Clin Cancer Res. 2021;40:328. 34663410 10.1186/s13046-021-02130-2 PMC8522158

[16] Areeb Z, Stuart SF, West AJ, Gomez J, Nguyen HPT, Paradiso L, et al. Reduced EGFR and increased miR-221 is associated with increased resistance to temozolomide and radiotherapy in glioblastoma. Sci Rep. 2020;10:17768. 33082482 10.1038/s41598-020-74746-x PMC7576591

[17] Salama Y, Hattori K, Heissig B. The angiogenic factor Egfl7 alters thymogenesis by activating Flt3 signaling. Biochem Biophys Res Commun. 2017;490:209–16. 28601636 10.1016/j.bbrc.2017.06.023

[18] Li T, Fan J, Wang B, Traugh N, Chen Q, Liu JS, et al. TIMER: a web server for comprehensive analysis of tumor-infiltrating immune cells. Cancer Res. 2017;77:e108–e10. 29092952 10.1158/0008-5472.CAN-17-0307 PMC6042652

[19] Liu H, Zhang B, Sun Z. Spectrum of EGFR aberrations and potential clinical implications: insights from integrative pan-cancer analysis. Cancer Commun (Lond). 2020;40:43–59. 32067422 10.1002/cac2.12005 PMC7163653

[20] Felli N, Errico MC, Pedini F, Petrini M, Puglisi R, Bellenghi M, et al. AP2α controls the dynamic balance between miR-126&126* and miR-221&222 during melanoma progression. Oncogene 2015;35:3016. 26434590 10.1038/onc.2015.357 PMC4908437

[21] van Solingen C, Seghers L, Bijkerk R, Duijs JMGJ, Roeten MK, van Oeveren-Rietdijk AM, et al. Antagomir-mediated silencing of endothelial cell specific microRNA-126 impairs ischemia-induced angiogenesis. J Cell Mol Med. 2009;13:1577–85. 19120690 10.1111/j.1582-4934.2008.00613.x PMC3828868

[22] Zeng W, Liu Y, Li W-T, Li Y, Zhu J-F. CircFNDC3B sequestrates miR-937-5p to derepress TIMP3 and inhibit colorectal cancer progression. Mol Oncol. 2020;14:2960–84. 32896063 10.1002/1878-0261.12796 PMC7607164

[23] Beck AC, Cho E, White JR, Paemka L, Li T, Gu VW, et al. AP-2α regulates S-phase and is a marker for sensitivity to PI3K inhibitor buparlisib in colon cancer. Mol Cancer Res. 2021;19:1156–67. 33753551 10.1158/1541-7786.MCR-20-0867 PMC8254761

[24] Wu S, Yuan W, Luo W, Nie K, Wu X, Meng X, et al. miR-126 downregulates CXCL12 expression in intestinal epithelial cells to suppress the recruitment and function of macrophages and tumorigenesis in a murine model of colitis-associated colorectal cancer. Mol Oncol. 2022;16:3465–89. 35363937 10.1002/1878-0261.13218 PMC9533691

[25] Li XM, Wang AM, Zhang J, Yi H. Down-regulation of miR-126 expression in colorectal cancer and its clinical significance. Med Oncol. 2011;28:1054–7. 20680522 10.1007/s12032-010-9637-6

[26] Almutairi S, Kalloush HM, Manoon NA, Bardaweel SK. Matrix metalloproteinases inhibitors in cancer treatment: an updated review (2013-2023). Molecules. 2023;28:5567. 10.3390/molecules28145567 PMC10384300 37513440

[27] Wang X, Fu X, Brown PD, Crimmin MJ, Hoffman RM. Matrix metalloproteinase inhibitor BB-94 (batimastat) inhibits human colon tumor growth and spread in a patient-like orthotopic model in nude mice. Cancer Res. 1994;54:4726–8. 8062271

[28] Xiao Y, Lian H, Zhong XS, Krishnachaitanya SS, Cong Y, Dashwood RH, et al. Matrix metalloproteinase 7 contributes to intestinal barrier dysfunction by degrading tight junction protein Claudin-7. Front Immunol. 2022;13:1020902. 10.3389/fimmu.2022.1020902 PMC9581388 36275703

[29] Han S, Li G, Jia M, Zhao Y, He C, Huang M, et al. Delivery of anti-miRNA-221 for colorectal carcinoma therapy using modified cord blood mesenchymal stem cells-derived exosomes. Front Mol Biosci. 2021;8:743013. 10.3389/fmolb.2021.743013 PMC8488275 34616773

[30] Ali A, Grillone K, Ascrizzi S, Caridà G, Fiorillo L, Ciliberto D, et al. LNA-i-miR-221 activity in colorectal cancer: a reverse translational investigation. Mol Ther Nucleic Acids. 2024;35:102221. 10.1016/j.omtn.2024.102221 PMC11168481 38868363

[31] Di Martino MT, Arbitrio M, Caracciolo D, Cordua A, Cuomo O, Grillone K, et al. miR-221/222 as biomarkers and targets for therapeutic intervention on cancer and other diseases: a systematic review. Mol Ther Nucleic Acids. 2022;27:1191–224. 35282417 10.1016/j.omtn.2022.02.005 PMC8891816

[32] Molnár V, Érsek B, Wiener Z, Tömböl Z, Szabó PM, Igaz P, et al. MicroRNA-132 targets HB-EGF upon IgE-mediated activation in murine and human mast cells. Cell Mol Life Sci. 2012;69:793–808. 21853268 10.1007/s00018-011-0786-3 PMC11114963

[33] Alzahrani, Hanieh AM, Ibrahim H, H-iM, Mohafez O, Shehata T, et al. Enhancing miR-132 expression by aryl hydrocarbon receptor attenuates tumorigenesis associated with chronic colitis. Int Immunopharmacol. 2017;52:342–51. 29017096 10.1016/j.intimp.2017.09.015

[34] Salama Y, Jaradat N, Hattori K, Heissig B. Aloysia citrodora essential oil inhibits melanoma cell growth and migration by targeting HB-EGF-EGFR signaling. Int J Mol Sci. 2021;22:8151. 10.3390/ijms22158151 PMC8347434 34360915

