# Supplementary material for: Heparin-binding EGF-like growth factor via miR-126 controls tumor formation/growth and the proteolytic niche in murine models of colorectal and colitis-associated cancers
Source: Cell Death Dis. 2024 Oct 17;15(10):753. doi: 10.1038/s41419-024-07126-2 (PMC11487245; doi:10.1038/s41419-024-07126-2)

Heparin-binding EGF-Like Growth Factor via miR-126 controls tumor formation/growth and the proteolytic niche in murine models of colorectal and colitis-associated cancers

Yousef Salama[1,2], Shinya Munakata[1,3], Taro Osada[4], Satoshi Takahashi[5], Koichi Hattori[6,7]* and Beate Heissig[1,8]*

Figure 2E

R1_Figure 2E

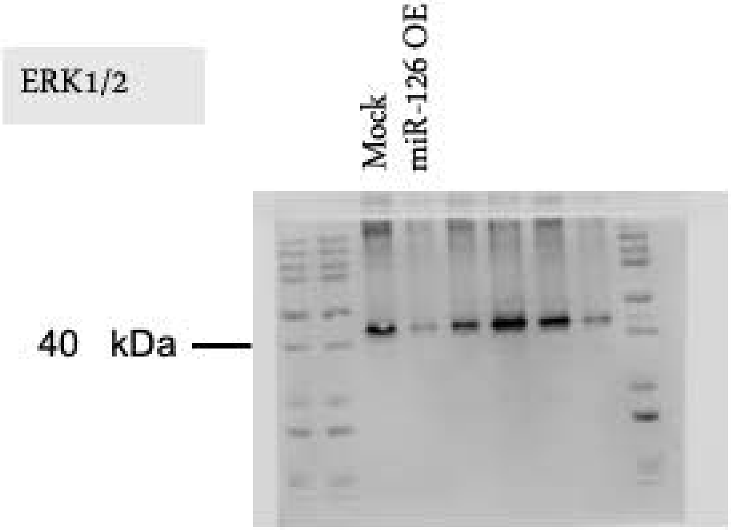

Original Blot Fig2E

published in
https://www.mdpi.com/1422-0067/22/15/8151

Salama et al. **Heparin-binding EGF-Like Growth Factor via miR-126 controls tumor formation and the proteolytic niche in murine models of colorectal and colitis-associated cancers.**
Fig. 2E

b-actin

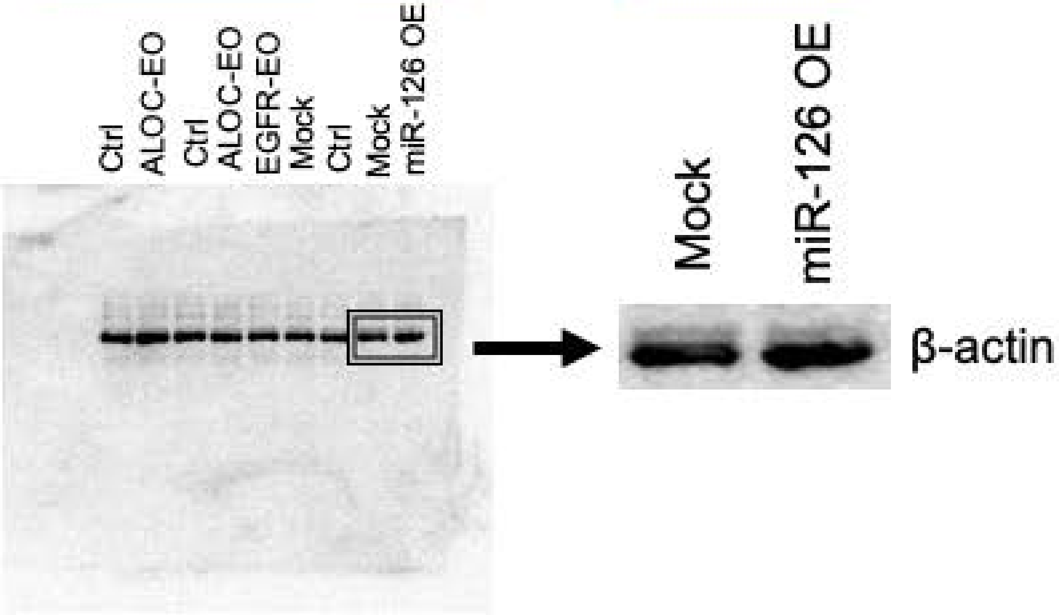

R1 Western blots

R1_Figure 3B and 4B

β-actin

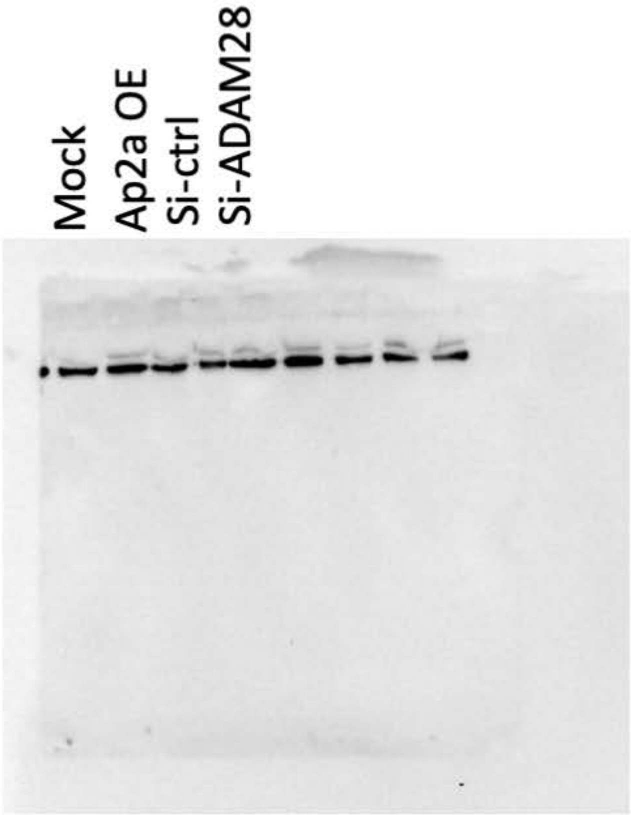

R1_Figure 3B

Ap2a OE

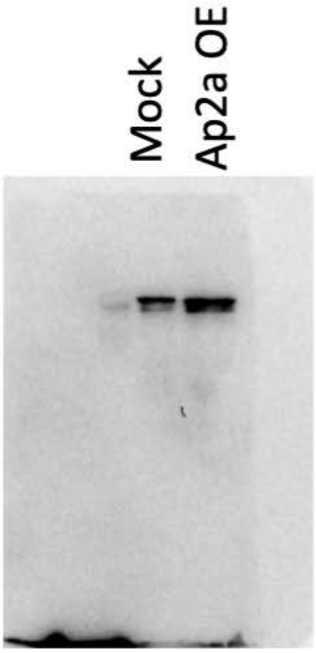

ADAM28

R1_Figure 4B

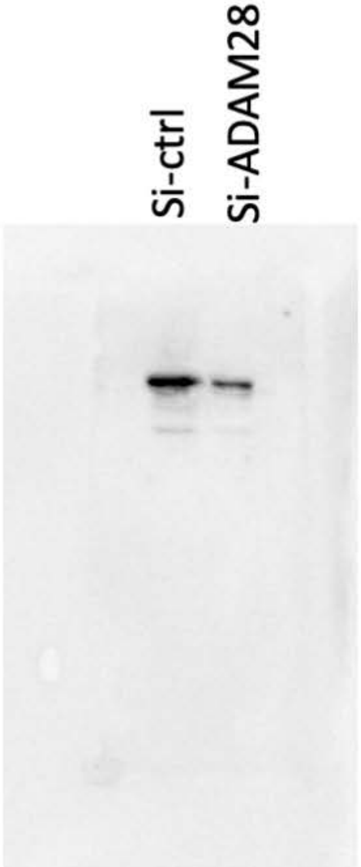

R1_Fig. 2E
R1_4E

Pro-HB-EGF

R1_Fig. 2E R1_Fig. 4E

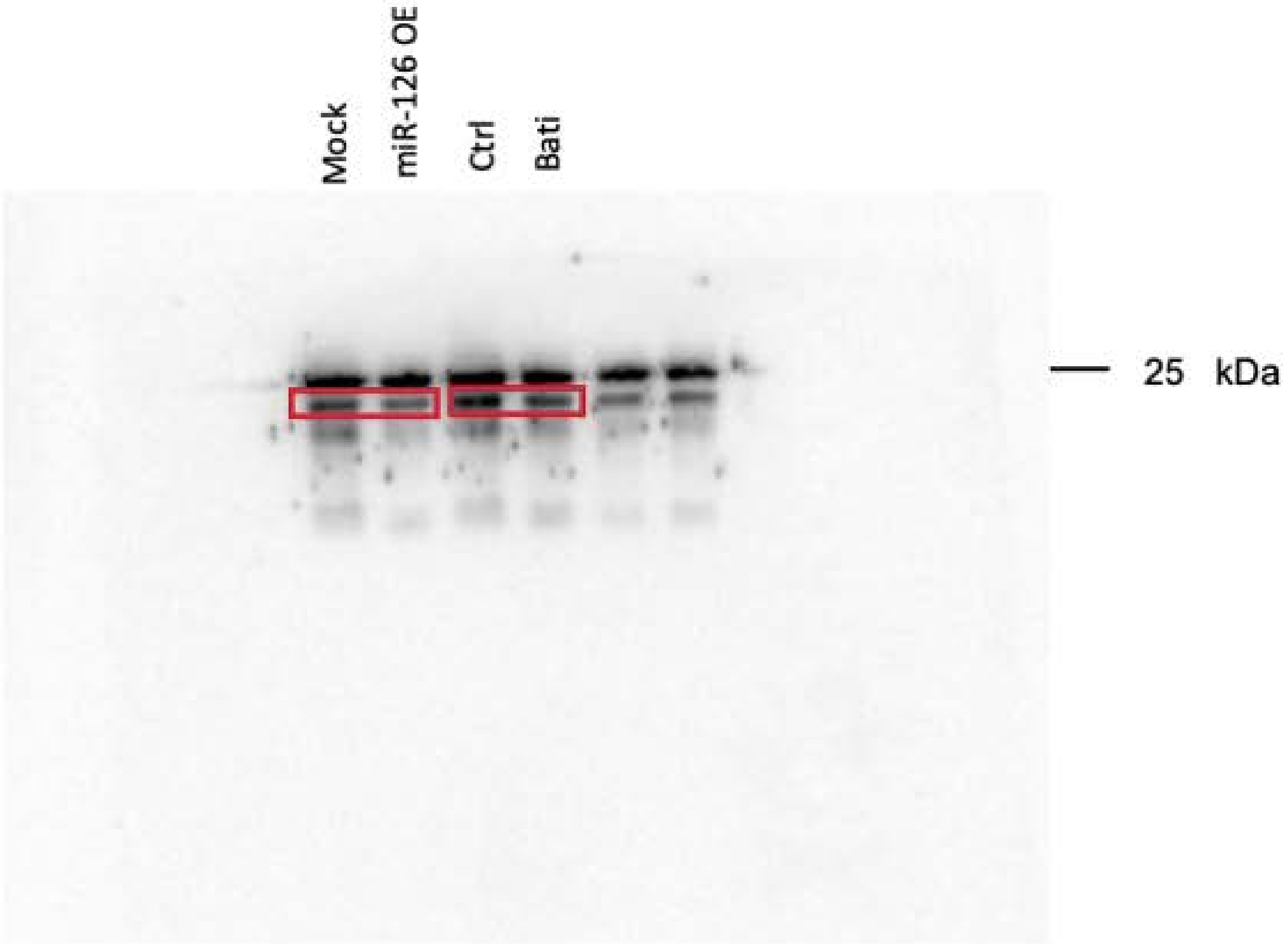

b-actin

R1_Fig4.E, lower panel

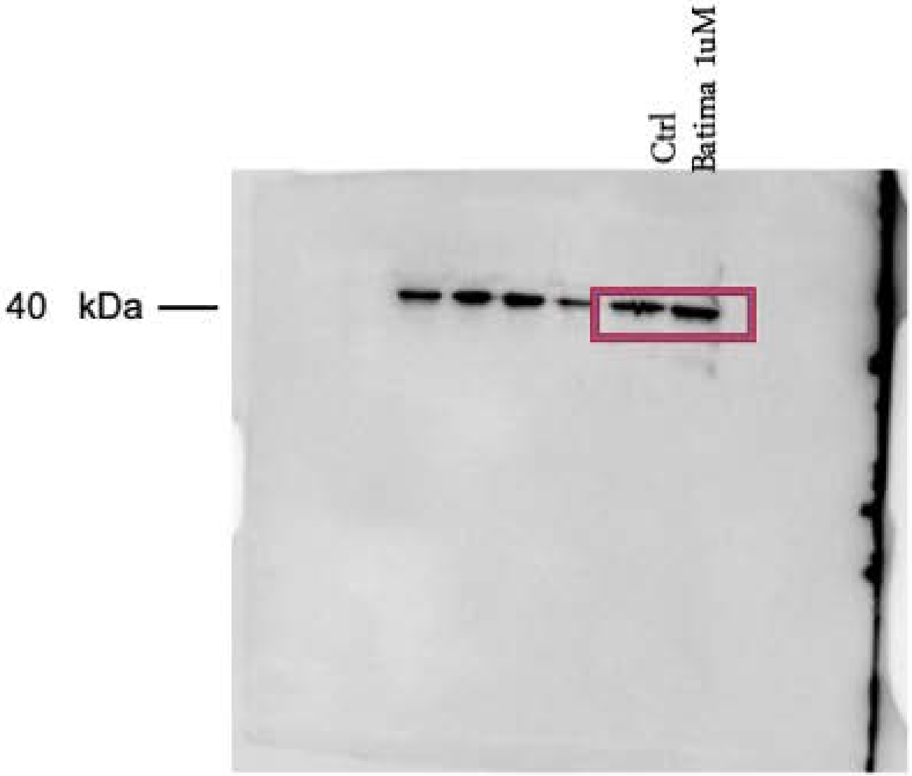

R1_Fig. 4B

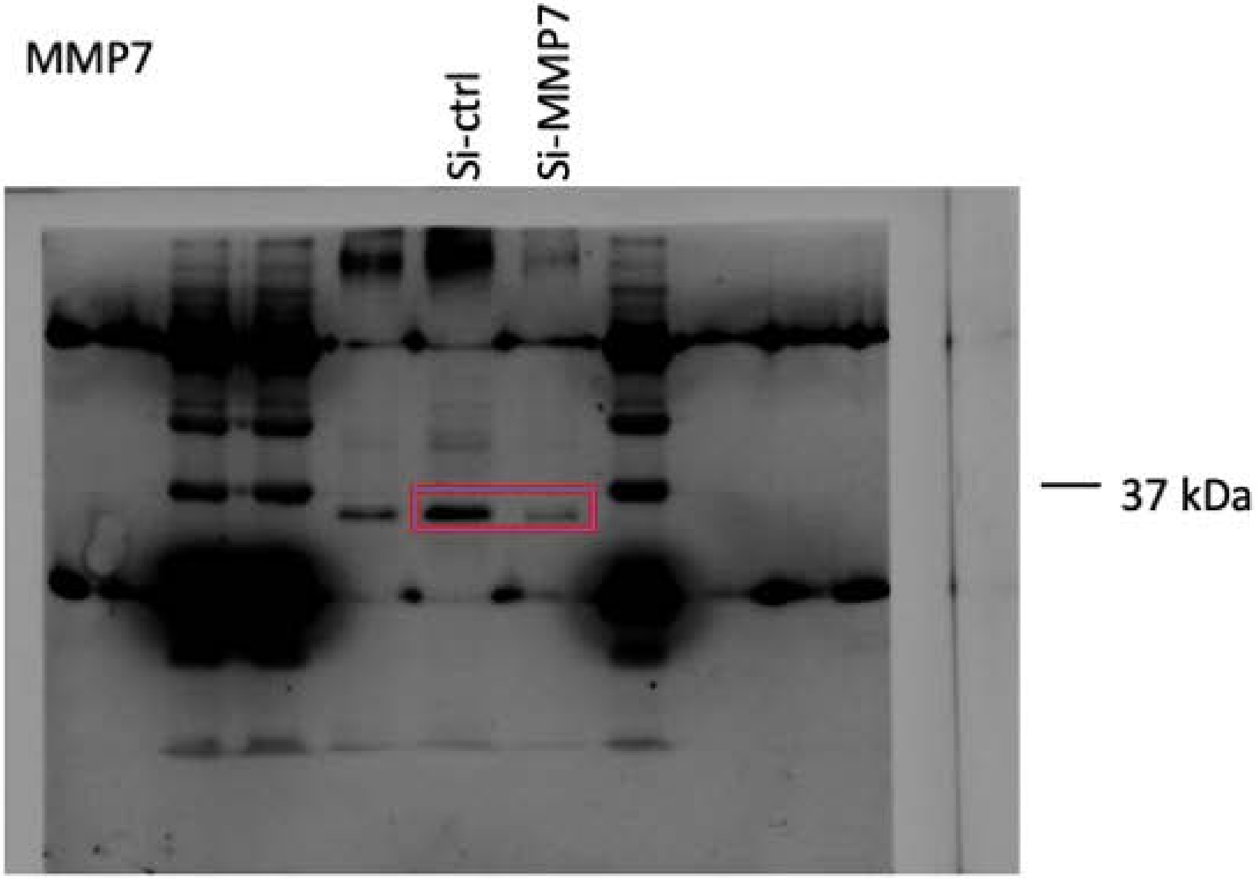

R1_Suppl. Figure 2A

R1_Suppl. Figure 2A

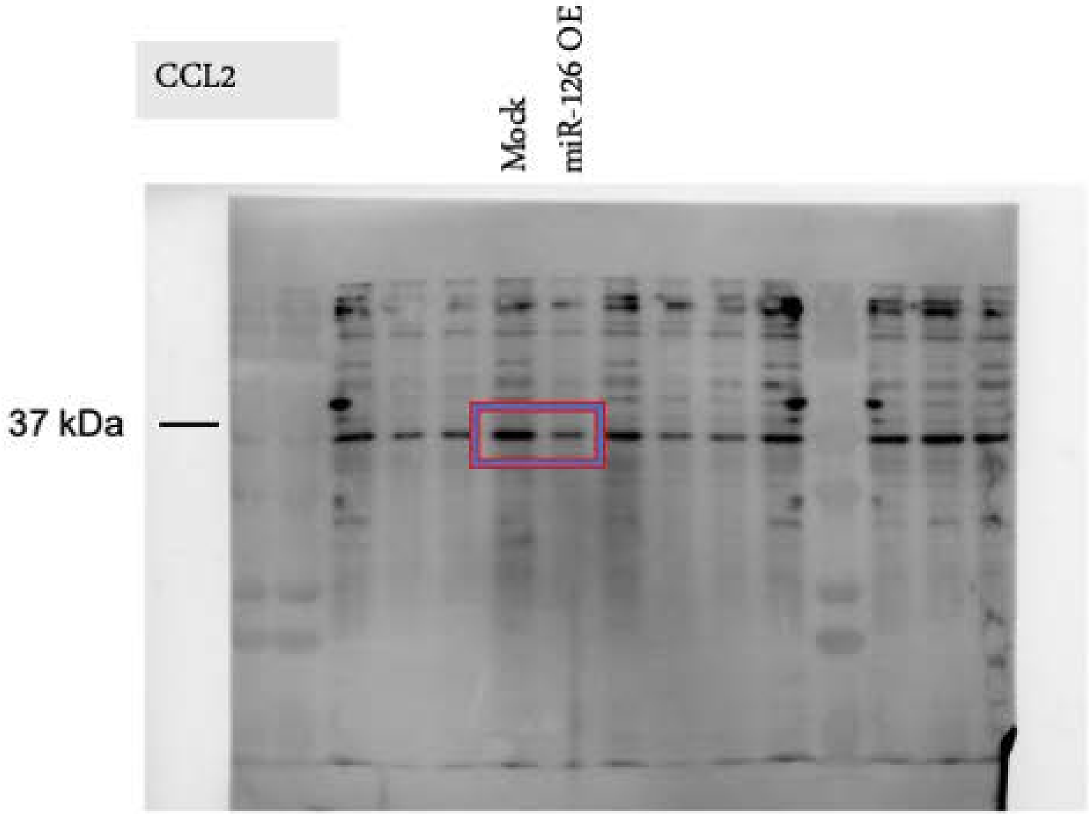

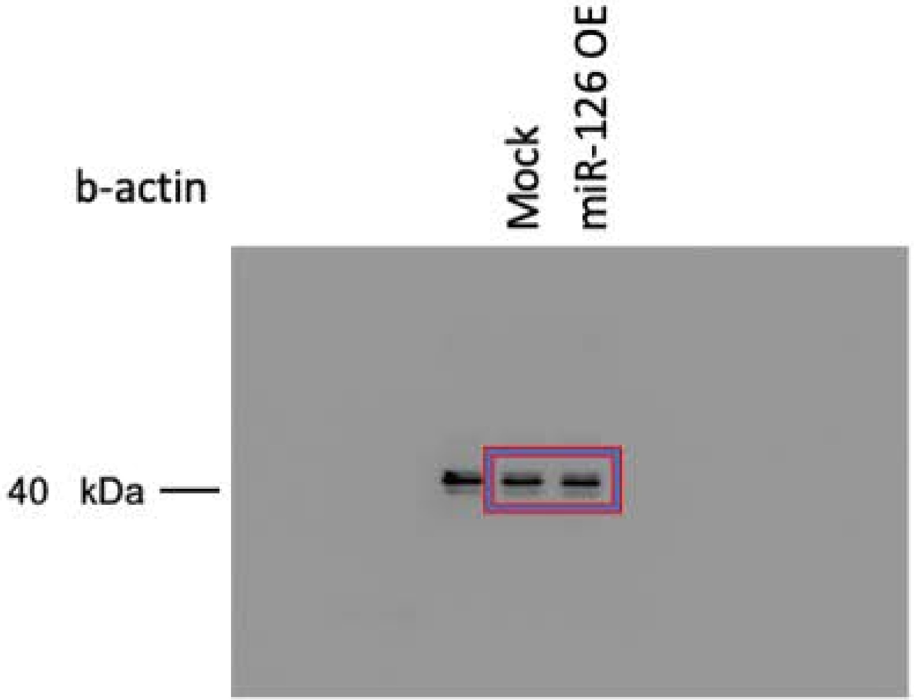

R1_Fig.S2D
R1_Fig4B

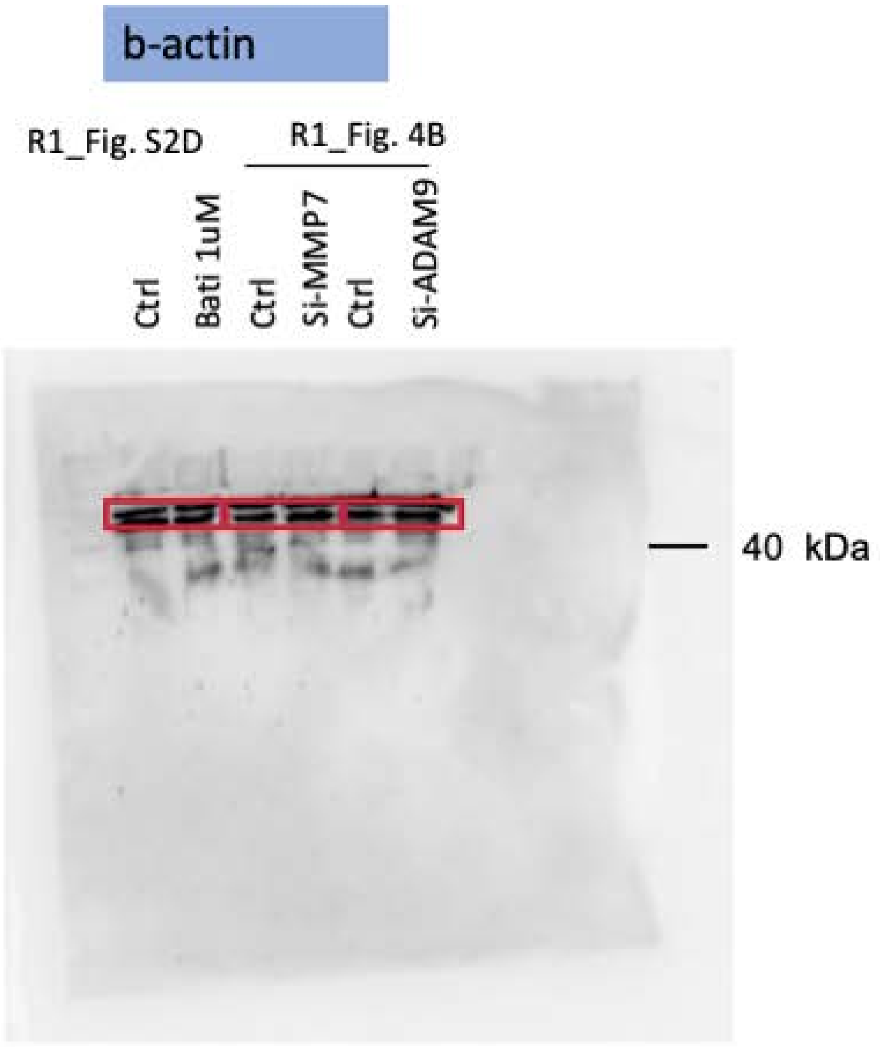

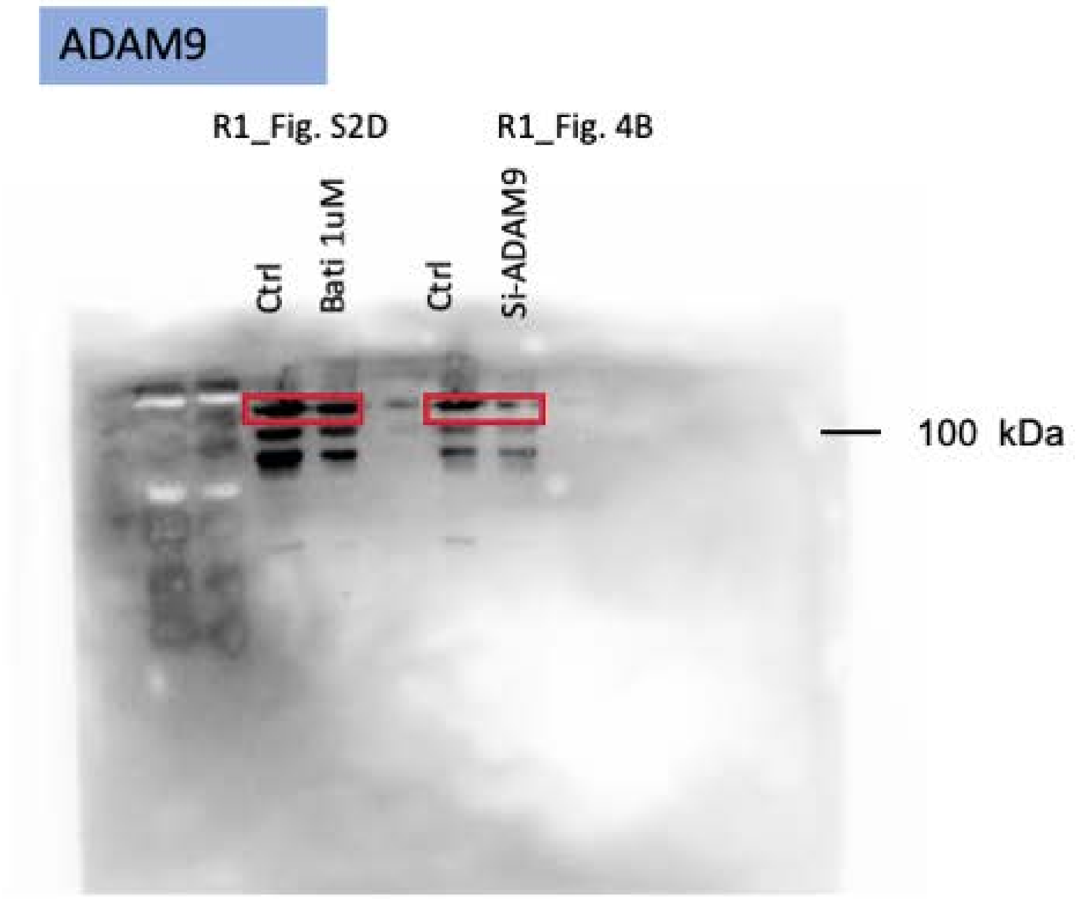

Fig.S2D

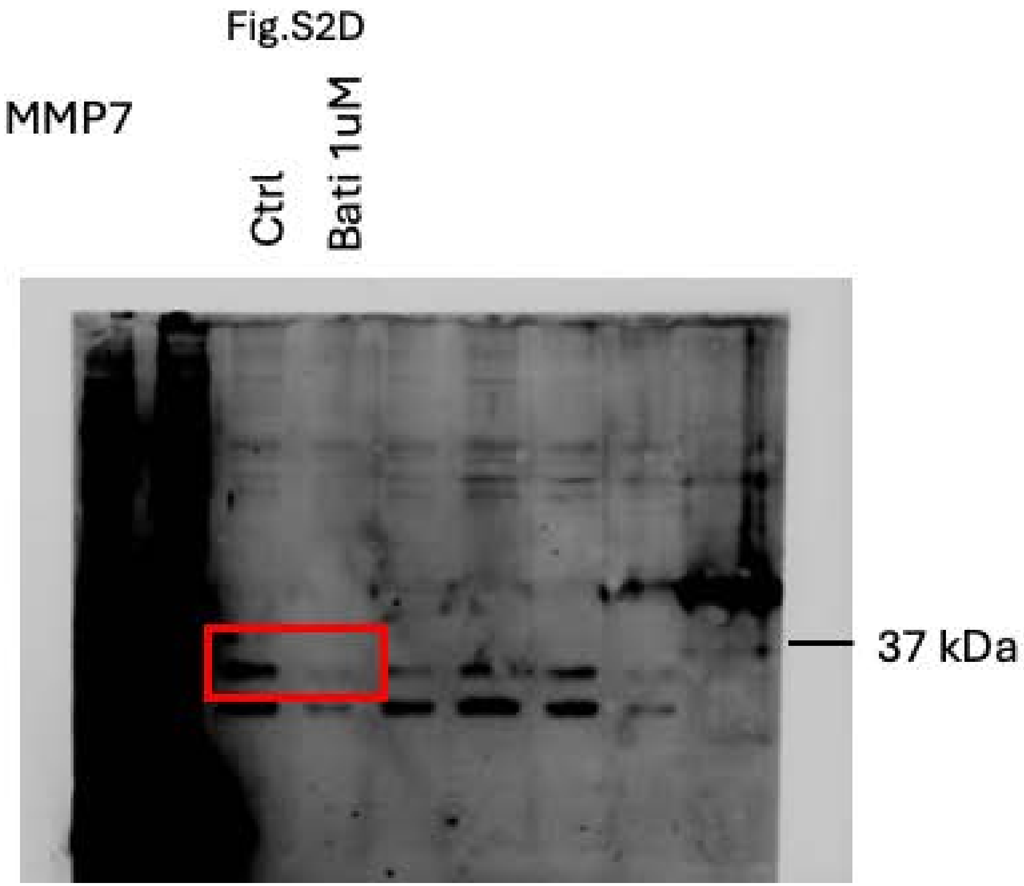

Supplement: Supplementary file 1 — Original western blots_All [file 41419_2024_7126_MOESM1_ESM.pdf]
