# Supplementary material for: Heparin-binding EGF-like growth factor via miR-126 controls tumor formation/growth and the proteolytic niche in murine models of colorectal and colitis-associated cancers
Source: Cell Death Dis. 2024 Oct 17;15(10):753. doi: 10.1038/s41419-024-07126-2 (PMC11487245; doi:10.1038/s41419-024-07126-2)
Supplement: Supplementary file 2 — FigureS1_2 [file 41419_2024_7126_MOESM2_ESM.pdf]

Supplementary Figures 1 and 2

Heparin-binding EGF-Like Growth Factor via miR-126 controls tumor formation/growth and the proteolytic niche in murine models of colorectal and colitis-associated cancers

Yousef Salama[1,2], Shinya Munakata[1,3], Taro Osada[4], Satoshi Takahashi[5], Koichi Hattori[6,7]* and Beate Heissig[1,8]*

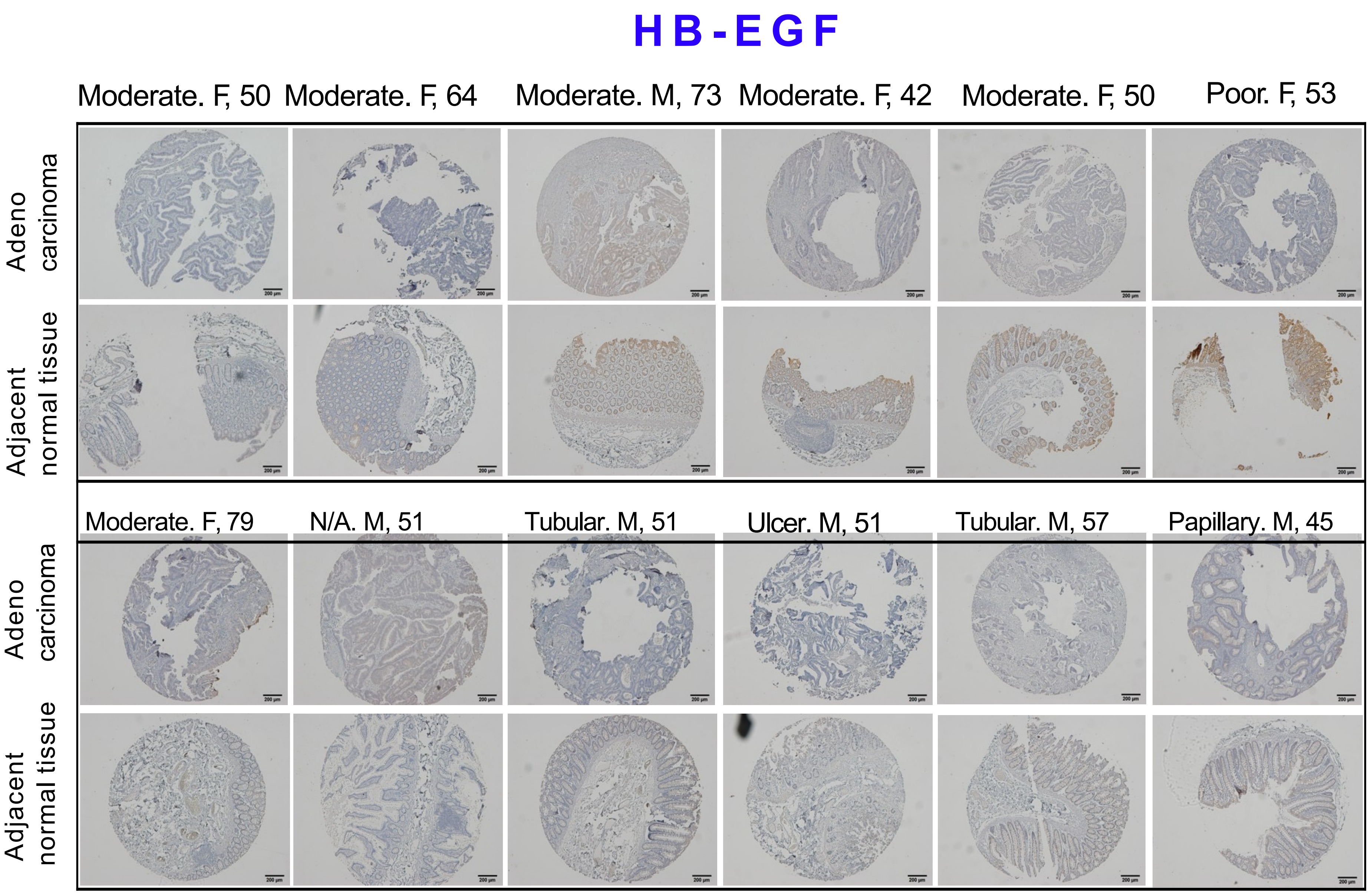

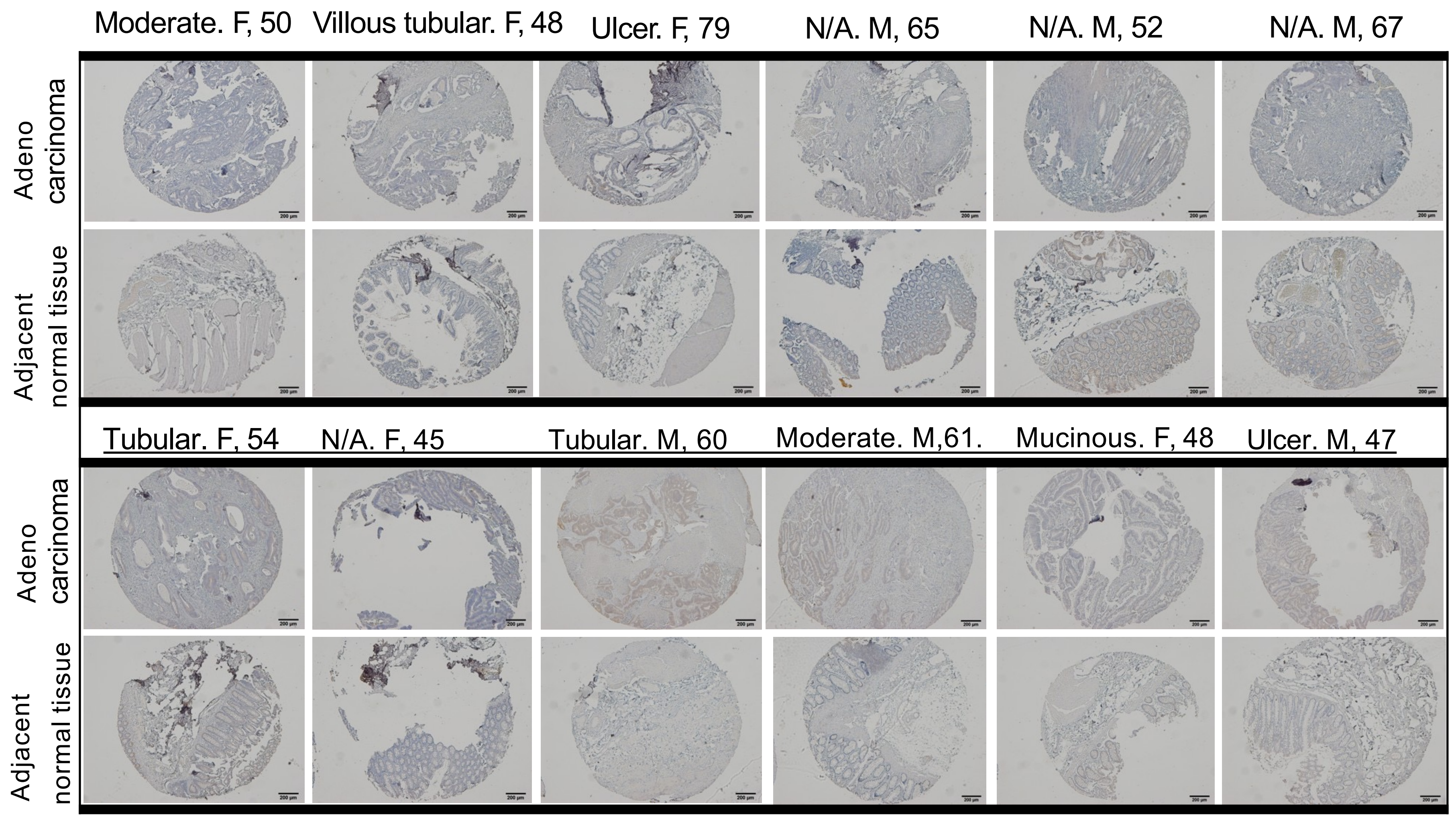

Supplementary data Figure 1S. Immunohistochemical detection of HB-EGF in tissue arrays from colon cancer and non-malignant adjacent areas of human patients. Representative images are given. Scale bar, 200 μm. The patient information is shown in the figure provided by the manufacturer.

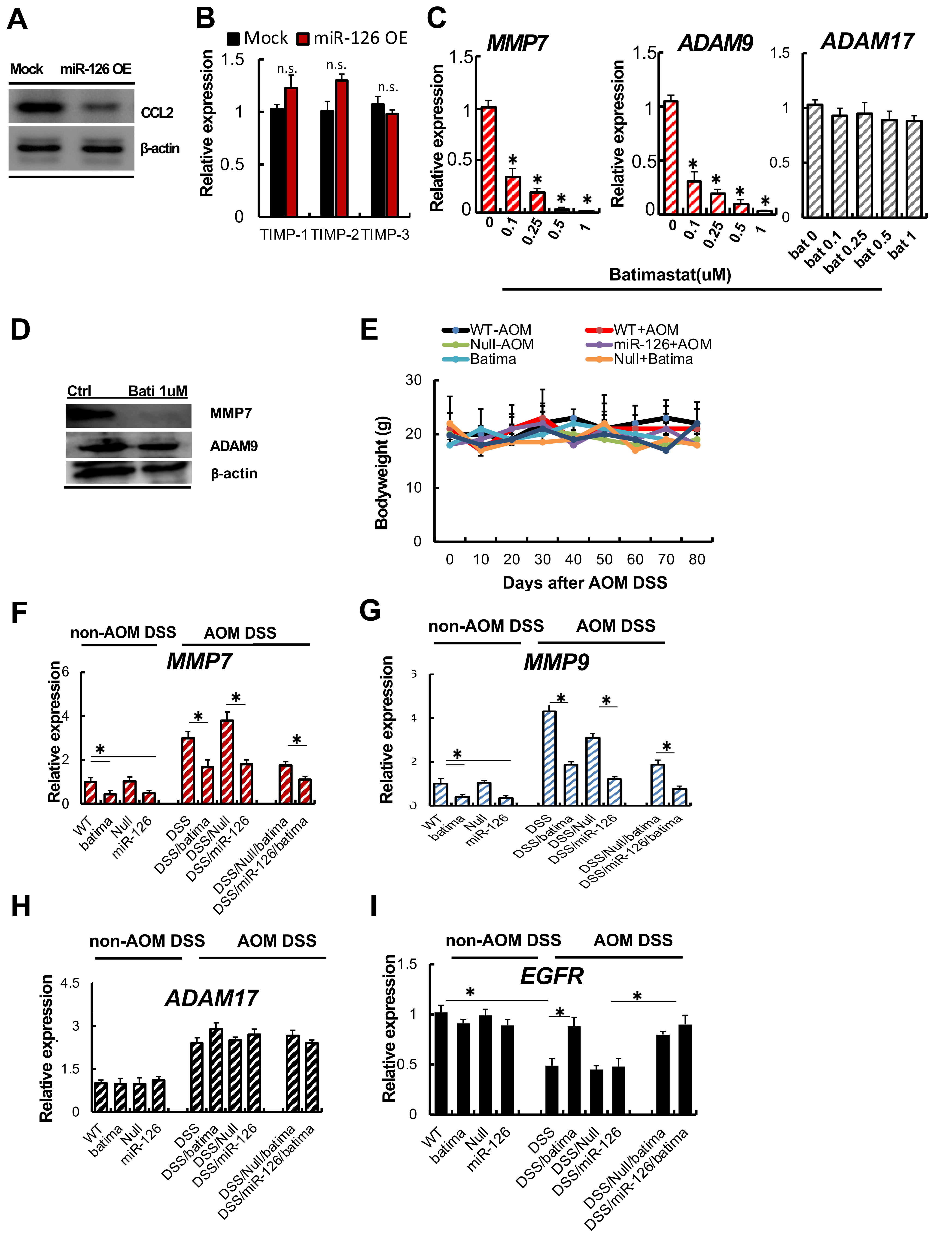

Supplementary data Figure 2. miR-126 OE and MMPi reduce MMP7/-9, but not ADAM17 gene expression. Representative immunoblot of CCL2 and the loading control b-actin of Mock and miR-126 OE CMT93 cells. B-actin and CCL-2 samples were run on separate gels. (B) Fold change in TIMP-1, TIMP-2, and TIMP-3 expression in miR-126 OE and ctrl CMT93 cells as determined by qPCR (n=3/group). (C) Fold change in MMP7, ADAM9, and ADAM17 expression in batimastat-treated compared to untreated CMT93 cells as determined by qPCR (n=3/group). (D) Representative immunoblot of ADAM9 (D) and b-actin of CMT93 cells treated with or without batimastat. Different gels for ADAM9 and b-actin. (E) Bodyweight in AOM DSS mice treated with AdNull, AdmiR-126, with or without batimastat (n=3/group). (F-I) AdmiR-126 or AdNull adenoviral injections and cotreatment with or without batimastat daily starting from day 20 in mice receiving AOM and DSS (AOM DSS mice) and mice not receiving AOM and DSS (non-AOM DSS mice). Colon tissues retrieved on day 80 were examined for MMP7 (F), MMP9 (G), ADAM17 (H), and EGFR (I) expression by qPCR. qPCR: Normalization of expression to b-actin. Data are presented as a relative fold change to untreated WT control without tumors according to the comparative Ct method (2−ΔΔCt.) and represented as mean ± SEM, with p values from unpaired Student's t-test. *p < .05, *** p < .001.
